# Adipocytes orchestrate obesity-related chronic inflammation through β2-microglobulin

**DOI:** 10.1038/s41392-025-02486-3

**Published:** 2025-12-03

**Authors:** Jie Li, Yuhao Li, Xiaoyang Zhou, Shushu Yang, Dong Liu, Hao Wen, Xiaoling Chen, Chengjie Duan, Meiling Yu, Mengjun Zhang, Bo Tang, Yong Wang, Li Wang, Yuzhang Wu

**Affiliations:** 1 Department of Immunology, College of Basic Medicine, Army Medical University (Third Military Medical University), Chongqing, China; 2 Department of Biosafety, College of Basic Medicine, Army Medical University (Third Military Medical University), Chongqing, China; 3 Department of Pharmaceutical Analysis, College of Pharmacy, Army Medical University (Third Military Medical University), Chongqing, China; 4 Department of General Surgery, The First Affiliated Hospital of Army Medical University, Chongqing, China; 5 Department of Laboratory Animal Science, College of basic medical sciences, Army Medical University, Chongqing, China

**Keywords:** Antigen processing and presentation, Immunological disorders, Inflammation

## Abstract

Chronic inflammation in adipose tissue is widely recognized as a pivotal link connecting obesity to a spectrum of related chronic diseases, including type 2 diabetes, non-alcoholic fatty liver disease, and cardiovascular disorders. In this pathogenic process, the dysregulated interaction between adipocytes and adipose-resident immune cells plays a critical regulatory role; however, the underlying mechanisms governing this abnormal interaction remain largely unknown. In this study, we showed that upregulated β2-microglobulin expression in hypertrophic adipocytes during obesity not only mediated the activation of adipose-resident CD8^+^ T cells in a cell contact-dependent manner but also facilitated iron overload and the ferroptosis of adipocytes, thereby promoting the M1 polarization of adipose tissue macrophages. Conversely, specific ablation of β2-microglobulin in adipocytes effectively suppressed the activation and accumulation of adipose-resident CD8^+^ T cells, as well as adipocyte ferroptosis and M1 polarization, ultimately preventing high-fat diet-induced obesity and its related inflammation and metabolic disorders. Additionally, adeno-associated virus-mediated adipose-targeted knockdown of β2-microglobulin has been demonstrated to therapeutically alleviate high-fat diet-induced obesity, as well as its related chronic inflammation and metabolic disorders. Furthermore, our bioinformatic analysis of human adipose transcriptome data revealed a strong correlation between adipose β2-microglobulin and obesity. More importantly, β2-microglobulin is significantly upregulated in adipocytes isolated from patients with obesity. Thus, our findings highlight the pivotal role of adipocytes in obesity-associated chronic inflammation and metabolic disorders via β2-microglobulin-dependent mechanisms.

## Introduction

Obesity is a chronic metabolic disease characterized by excessive fat accumulation and/or abnormal adipose tissue distribution in the body.^1^ It has emerged as a leading global public health concern and serves as a common pathophysiological foundation for numerous chronic diseases.^2^ With an estimated overweight prevalence of nearly 50% among the world’s adult population, obesity has emerged as a major global public health challenge in recent years.^1^ Worryingly, a high body mass index (BMI, ≥25) is increasingly recognized as a prominent risk factor for a wide range of non-communicable diseases, including metabolic diseases (e.g., Type 2 diabetes mellitus, T2DM; metabolic dysfunction-associated fatty liver disease, MAFLD), cardiovascular diseases (e.g., hypertension, coronary heart disease, and stroke), respiratory diseases (e.g., asthma and obstructive sleep apnea syndrome), malignancies (e.g., breast and colorectal cancer), and mental health disorders (e.g., depression and anxiety).^1–3^ Chronic inflammation of adipose tissue represents a persistent, low-grade, systemic inflammatory condition that originates in adipose tissue, characterized by excessive lipid accumulation. It can progressively affect multiple organs, including the liver, skeletal muscle, pancreatic islets, and the central nervous system, and plays a pivotal role in the pathogenesis of obesity-related metabolic complications.^4–6^ Therefore, elucidating the underlying initiation mechanisms driving chronic adipose tissue inflammation is vital for the effective prevention and management of metabolic syndrome associated with obesity.^6–8^ However, our current understanding of the regulatory mechanisms underlying these inflammatory cascades remains insufficient.

The composition of adipose tissue is intricate, with one-third being mature adipocytes, and the remaining components include preadipocytes, fibroblasts, endothelial cells, and immune cells, collectively referred to as the stromal vascular fraction (SVF).^9,10^ Immune cells residing within adipose tissue have been implicated as key players in the pathogenesis of obesity-related inflammation and metabolic disorders.^10,11^ Early research on adipose tissue-resident immune cells has identified adipose tissue macrophages (ATMs) as central mediators of inflammatory responses.^12,13^ As the most abundant immune cells in adipose tissue, ATMs are dynamically regulated under obese pathological conditions: for instance, the CC motif ligand-2/chemokine receptor type 2 chemokine axis mediates the infiltration of circulating monocytes into obese adipose tissue.^14^ These infiltrating monocytes serve as an important cellular source for ATMs, which, together with the massive proliferation of ATMs, contribute to a substantial increase in ATM accumulation. Concurrently, ATMs undergo a phenotypic shift from the anti-inflammatory M2 state to the pro-inflammatory M1 phenotype, which drives obesity-associated chronic inflammation and metabolic disorders.^10,15^ M1 macrophages can secrete high levels of pro-inflammatory cytokines, including interferon γ (IFNγ), and interleukin-1β (IL-1β), thereby exacerbating adipose tissue inflammation and promoting systemic low-grade inflammation via circulation, ultimately contributing to metabolic dysfunction.^10,15^ Despite growing recognition of the pivotal role of ATMs in obesity-induced metabolic complications, our understanding of the mechanisms governing the inflammatory activation of ATMs under obese conditions remains incomplete and unclear.

In recent years, the role of adipose-resident T cells in regulating chronic inflammation of adipose tissue associated with obesity has become increasingly well-established, offering novel insights into this area of research.^10,15^ For instance, CD8⁺ T cells are among the earliest activated immune cells in adipose tissue during the initiation of obesity and play a pivotal role in the early stages of obesity-related inflammatory responses.^16^ On one hand, they secrete pro-inflammatory cytokines that not only promote the recruitment of ATMs to adipose tissue but also induce the polarization of ATMs toward the M1 type; on the other hand, they can directly participate in the amplification of inflammatory signals, collectively positioning them as key drivers of obesity-related chronic inflammation.^17^ Although the core role of adipose-resident immune cells in obesity-related chronic inflammation and metabolic disorders has been clearly confirmed, the molecular mechanisms that drive the phenotypic remodeling and inflammatory activation of these immune cells under obese conditions remain not fully clarified.

Adipocytes are not only responsible for the storage and supply of nutrients but also play a crucial role in regulating energy balance throughout the body.^18,19^ Hypertrophic adipocytes are believed to be closely associated with chronic inflammation and metabolic disorders related to obesity. In obesity, hypertrophic adipocytes experience hypoxia stress, endoplasmic reticulum stress, or dysregulation of multiple metabolic pathways, such as creatine phosphate/creatine and glutamine metabolism, contributing to the development of obesity-related chronic inflammation, largely through interactions with adipose-resident immune cells.^20–22^ However, the exact role and mechanisms of hypertrophic adipocytes in the inflammatory activation of adipose-resident immune cells remain incompletely understood.

Here, we discovered a previously unrecognized role of adipocytes in the inflammatory activation of adipose-resident immune cells to promote obesity-associated chronic inflammation and metabolic disorders, which is mediated through β2 microglobulin (B2M)-dependent mechanisms involving major histocompatibility complex class I (MHC-I) antigen presentation and iron transport. We believe that these findings not only significantly advance our understanding of chronic adipose inflammation driven by dysregulated adipocytes but also offer a potential novel therapeutic target to control obesity and its metabolic complications.

## Results

### B2M is upregulated in hypertrophic adipocytes

**Fig. 1:**
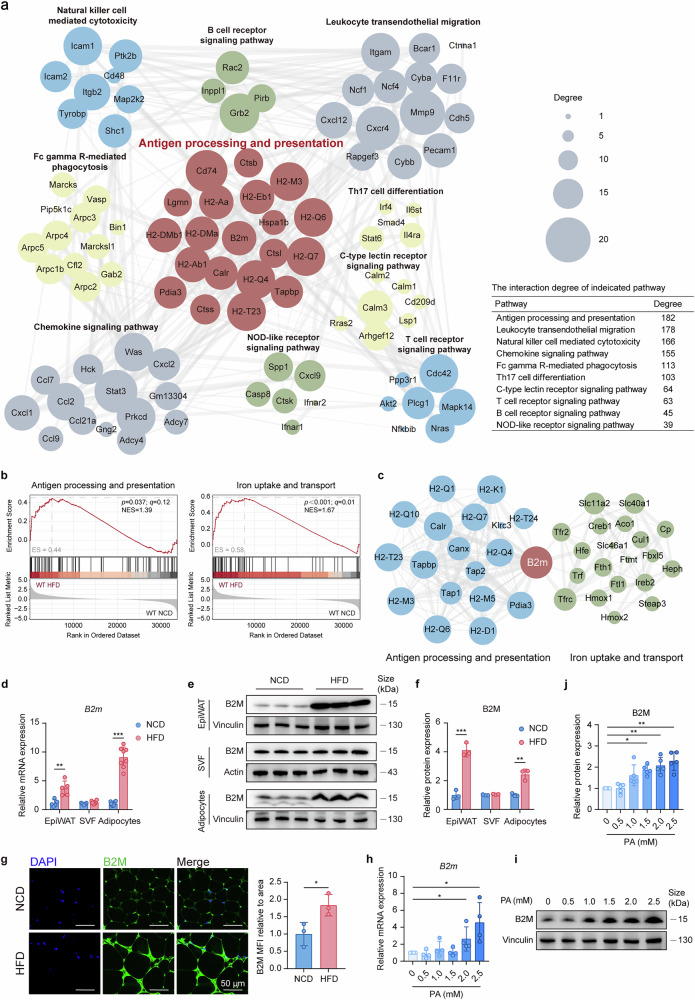
B2M is upregulated in hypertrophic adipocytes. **a**–**c** A transcriptome analysis of mature adipocytes isolated from EpiWAT of C57BL/6J mice on 16-week NCD and HFD. **a** Network diagram illustrating the interaction among the top 10 immune-related pathways enriched by KEGG analysis of DEGs. The table showing the degree of interaction for each pathway, as assessed by the cumulative interaction scores of the genes within the pathway. **b** GSEA of DEGs showing two B2M-associated pathways in adipocytes. **c** Network diagram illustrating the interaction among genes in antigen processing and presentation pathway (blue) and iron uptake and transport pathway (green), with B2M (red) at the intersection. Relative *B2m* mRNA levels (**d**), protein expressions (**e**) and relative quantification (**f**) in EpiWAT, SVF and purified mature adipocyte fraction from EpiWAT of 16-week NCD- or HFD-fed mice (*n* = 3-8). **g** Representative fluorescence images of B2M (green) and DAPI (blue) staining in EpiWAT sections of NCD-fed (upper) and HFD-fed (below) mice. The right panel quantifies the area-normalized B2M MFI (n = 3). Scale bar = 50 μm. Relative *B2m* mRNA levels (**h**), protein expressions (**i**) and relative quantification (**j**) in 3T3-L1 adipocytes treated with PA at the indicated dose for 24 h (*n* = 4-5). The data are representative as the mean ± standard deviation (SD), with “n” representing the number of biological replicates per experimental group. Significant in **d**, **f**, and **g** was calculated using a two-tailed Student’s *t* test. Significant in **h** and **j** was calculated using one-way ANOVA followed by Tukey’s HSD post hoc test for multiple comparisons. *p < 0.05, **p < 0.01, ***p < 0.001. B2M β2-microglobulin, DEGs differentially expressed genes, EpiWAT epididymal adipose tissue, HFD high-fat diet, MFI mean fluorescence intensity, NCD normal chow diet, PA palmitic acid, SVF stromal vascular fraction

To explore the potential contribution of adipocytes to obesity-related inflammation and metabolic disorders, we conducted a comparative transcriptome analysis of mature adipocytes purified from the epididymal adipose tissue (EpiWAT) of lean mice fed a normal chow diet (NCD) and obese mice fed a high-fat diet (HFD) (Supplementary Fig. 1a–l). Our analysis revealed 10,385 differentially expressed genes (DEGs), with 6,563 genes upregulated and 3822 genes downregulated in adipocytes from obese mice compared with lean mice (Supplementary Fig. 1m). Kyoto Encyclopedia of Genes and Genomes (KEGG) enrichment analysis of the DEGs revealed significant enrichment in 18 immune-related pathways (Supplementary Fig. 1n). Analysis of the interaction network of DEGs from the top 10 immune-related pathways indicated that the antigen processing and presentation pathway presented the highest degree of interaction, positioning it at the center of the network (Fig. 1a). Some DEGs within this pathway are involved in major histocompatibility complex class II (MHC-II)-related antigen presentation, which aligns with previous findings that hypertrophic adipocytes can function as antigen-presenting cells (APCs), directly activating CD4^+^ T cells via the enhanced MHC-II pathway in obesity.^23^ However, another set of genes, whose role in obesity has received little attention, is associated with MHC-I-related antigen presentation. Among them, B2M, a critical chaperone for MHC-I molecules, has attracted our interest. Notably, in addition to the antigen processing and presentation pathway, the *B2m* gene is also involved in the iron uptake and transport pathway, both of which are significantly upregulated in adipocytes from obese mice (Fig. 1b). Importantly, the *B2m* gene seemed to be the intersection connecting the interaction network of these two pathways (Fig. 1c). Validation experiments confirmed substantial upregulation of B2M expression at both the mRNA and protein levels in EpiWAT from obese mice compared with lean mice (Fig. 1d–f), whereas no such changes were observed in other metabolically relevant tissues, such as the liver and skeletal muscle (Supplementary Fig. 1o, p). Moreover, the upregulation of B2M in the EpiWAT of obese mice was attributed to mature adipocytes rather than the SVF (Fig. 1d–f). Consistently, immunofluorescence staining also confirmed the increased expression of B2M in hypertrophic adipocytes from HFD-fed mice (Fig. 1g). Additionally, we observed a dose-dependent increase in both the mRNA and protein expression of B2M in hypertrophic 3T3-L1 adipocytes treated with palmitic acid (PA) in vitro (Fig. 1h–j). Collectively, these findings imply that B2M might play a significant role in the response of adipocytes to HFD-induced obesity.

### Adipocyte-specific B2M deletion protects mice from obesity and related inflammation and metabolic disorders

To assess the role of B2M in adipocytes in HFD-induced obesity, we generated adipocyte-specific B2M knockout (*B2m*^cKO^) mice (Supplementary Fig. 2a). *B2m*^cKO^ mice selectively ablated B2M in adipocytes (Supplementary Fig. 2b–e), whereas B2M expression in other tissues and the SVF was unaffected (Supplementary Fig. 2f-i). Under NCD conditions, no obvious differences were observed in body shape, weight gain, body composition, organ weight or histology between *B2m*^cKO^ mice and *B2m*^f/f^ mice (Supplementary Fig. 2j–o). Similarly, adipocyte-specific B2M ablation had no significant effect on glucose tolerance or insulin sensitivity or on serum adipocytokine levels, lipid parameters, or inflammatory cytokines (Supplementary Fig. 2p–t). Collectively, these results suggest that B2M ablation in adipocytes does not significantly alter the metabolic phenotype of mice under NCD conditions.

**Fig. 2:**
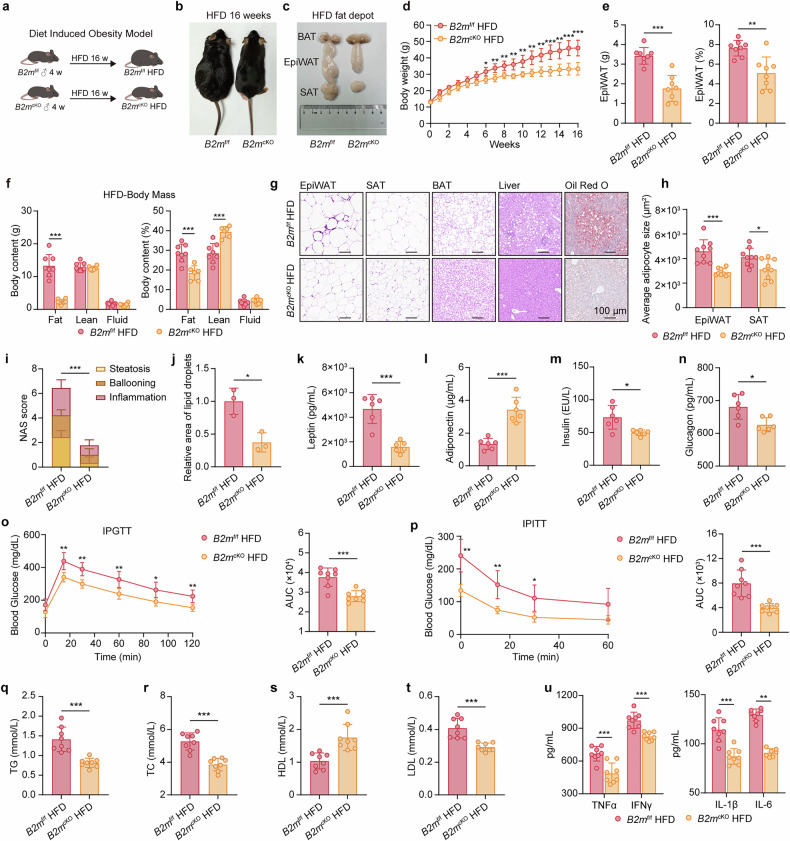
Adipocyte-specific B2M deletion protects mice from obesity and related inflammation and metabolic disorders. **a** Study design of diet induced obesity model. **b**, **c** Representative photograph of *B2m*^f/f^ and *B2m*^cKO^ mice and their respective fat pads. **d** Body weights (*n* = 8). **e** Weight and proportion of EpiWAT (*n* = 8). **f** Body content (fat, lean, water) analyzed by NMR spectrometer (*n* = 6–8). Representative histological images showing H&E staining and Oil Red O staining of indicated sections of HFD-fed *B2m*^f/f^ and *B2m*^cKO^ mice (**g**), with quantitation of average adipocyte size of EpiWAT and SAT section (**h**), NAS (**i**), and quantitation of Oil Red O staining (**j**) (*n* = 3). Scale bars, 100 μm. **k**–**n** Serum levels of leptin, adiponectin, insulin, and glucagon in HFD-fed *B2m*^f/f^ and *B2m*^cKO^ mice after 4 hours of fasting (*n* = 6). **o**, **p** IPGTT (2 g glucose per kg body weight), IPITT (0.75 U insulin per kg body weight) and their respective AUC (*n* = 8). **q**–**t** Serum levels of TG, TC, HDL and LDL (*n* = 8). **u** Serum levels of TNFα, IFNγ, IL-1β, and IL-6 (*n* = 7-8). The data are represented as the mean ± standard deviation (SD), with “n” representing the number of biological replicates per experimental group. Significance in **e**, **f**, **h**, **j**–**n**, AUC in **o**, AUC in **p**, and **q**–**u** was calculated using a two-tailed Student’s *t* test. Significance in **d**, **o**, and **p** was calculated using two-way ANOVA followed by Tukey’s HSD post hoc test for multiple comparisons. Significance in **i** was calculated using the Mann-Whitney *U* test. *p < 0.05, **p < 0.01, ***p < 0.001. AUC area under the curve, BAT brown adipose tissue, EpiWAT epididymal adipose tissue, HDL high density lipoprotein, HFD high-fat diet, IPGTT intraperitoneal glucose tolerance test, IPITT intraperitoneal insulin tolerance test, LDL low density lipoprotein, NAS Non-alcoholic fatty liver disease activity score, NCD normal chow diet, SAT subcutaneous adipose tissue, TC cholesterol, TG triglyceride

We then subjected both *B2m*^cKO^ and *B2m*^f/f^ mice to HFD challenge (Fig. 2a). With comparable food consumption, *B2m*^cKO^ mice presented significantly lower body weights and less EpiWAT gain, smaller adipocyte sizes in EpiWAT and subcutaneous adipose tissue (SAT), and reduced accumulation of lipid droplets in brown adipose tissue (BAT) than did *B2m*^f/f^ mice (Fig. 2b–h, Supplementary Fig. 2m, u). Furthermore, adipocyte-specific B2M ablation significantly decreased the number of crown-like structures (composed of ATMs surrounding dead or dying adipocytes) in EpiWAT and mitigated hepatic lipid accumulation (Fig. 2g–j). Notably, *B2m*^cKO^ mice also exhibited improved glucose tolerance and insulin sensitivity, ameliorated dysregulation of adipocytokine secretion and lipid disorders, and reduced circulating inflammatory cytokine levels (Fig. 2k–u). Collectively, these results strongly indicate a pivotal role of B2M in adipocytes in driving obesity and related inflammation and metabolic disorders.

### Adipocyte-specific B2M deletion hinders the HFD-induced accumulation of inflammatory immune cells in adipose tissue

Next, to investigate the regulatory effect of B2M on adipocytes during obesity and its potential mechanisms, we conducted a comparative analysis of the RNA-seq data obtained from mature adipocytes in the EpiWAT of control and *B2m*^cKO^ mice fed a HFD (Supplementary Fig. 3a). This analysis revealed 6,068 DEGs, with 849 genes upregulated and 5,219 genes downregulated in *B2m*^cKO^ mice compared with control mice under HFD conditions (Supplementary Fig. 3b). Gene Ontology (GO) and KEGG enrichment analyses revealed that the DEGs were enriched primarily in pathways related to inflammation activation, adipokine and chemokine signaling, antigen processing and presentation, ferroptosis, and iron homeostasis (Supplementary Fig. 3c, d). Further gene set enrichment analysis (GSEA) indicated that metabolic pathways, including glutathione metabolism, fatty acid beta-oxidation, oxidative phosphorylation of fatty acids, and PPAR signaling, were significantly upregulated in B2M-deleted adipocytes under HFD conditions, suggesting improved metabolic functions (Supplementary Fig. 3e, f). Moreover, pathways involved in T-cell activation, antigen presentation, and inflammatory responses, as well as macrophage activation, were significantly downregulated, indicating a reduction in inflammatory processes (Supplementary Fig. 3e, f).

**Fig. 3:**
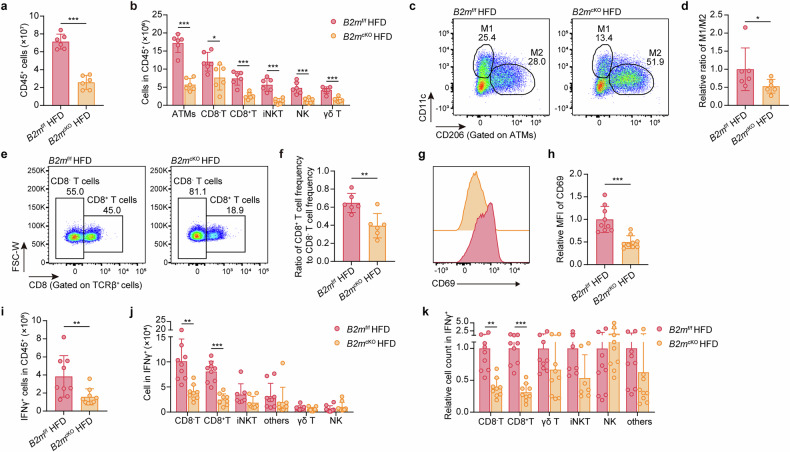
Adipocyte-specific B2M deletion hinders the HFD-induced accumulation of inflammatory immune cells in adipose tissues. **a** The absolute number of CD45^+^ cells in the EpiWAT (*n* = 6). **b** The absolute number of CD11b^+^F4/80^+^ ATMs, CD8^-^ T cells, CD8^+^ T cells, iNKT cells, NK cells, and γδ T cells within CD45^+^ cells in (**a**) (*n* = 4–6). Representative flow cytometry plots (**c**) and relative ratio (**d**) of CD11c^+^CD206^-^ M1 and CD11c^-^CD206^+^ M2, gated on CD11b^+^F4/80^+^ ATMs in the EpiWAT (*n* = 6). Representative flow cytometry plots (**e**) and ratio (**f**) of CD8^-^ T cell frequency and CD8^+^ T cell frequency, gated on CD3^+^TCRβ^+^ cells, in the EpiWAT (*n* = 6). Representative flow cytometry histograms (**g**) and relative MFI (**h**) of CD69 expression in CD8^+^ T cells in the EpiWAT (*n* = 9). **i** The absolute number of IFNγ^+^ cells (*n* = 9). The absolute number (**j**) and relative cell count (**k**) of indicated cells (*n* = 7–9). The data are representative as the mean ± standard deviation (SD), with “n” representing the number of biological replicates per experimental group. Significant in **a**, **b**, **d**, **f**, **h**, and **i**–**k** was calculated using a two-tailed Student’s *t* test. *p < 0.05, **p < 0.01, ***p < 0.001. ATMs, adipose tissue macrophages, EpiWAT epididymal adipose tissue, HFD high-fat diet, iNKT invariant Natural Killer T cell, MFI Mean fluorescence intensity, NK Natural Killer

Then, we carried out flow cytometric analyses to assess the number of EpiWAT-resident immune cells in HFD-fed *B2m*^f/f^ and *B2m*^cKO^ mice (Supplementary Fig. 4). As expected, the number of CD45^+^ immune cells in EpiWAT was significantly lower in HFD-fed *B2m*^cKO^ mice than in HFD-fed *B2m*^f/f^ mice (Fig. 3a). CD11b^+^F4/80^+^ ATMs accounted for the largest proportion of EpiWAT-infiltrated CD45^+^ immune cells, followed by CD3^+^TCRb^+^CD8^-^ T cells and CD3^+^TCRb^+^CD8^+^ T cells, with relatively fewer iNKT, NK and γδ T cells (Fig. 3b). Adipocyte-specific B2M ablation resulted in varying degrees of reduction in the number of these populations in the EpiWAT of HFD-fed *B2m*^cKO^ mice, with ATMs and CD8^+^ T cells showing nearly a two-thirds reduction in absolute number (Fig. 3b). Strikingly, the M1/M2 ratio and CD8^+^ T-cell/CD8^-^ T-cell ratio in the EpiWAT of HFD-fed *B2m*^cKO^ mice were significantly lower than those in the EpiWAT of HFD-fed control mice, indicating that adipocyte-specific B2M loss led to a preferential reduction in M1 and CD8^+^ T cells in adipose tissue (Fig. 3c–f). Additionally, adipose-resident CD8^+^ T cells from HFD-fed *B2m*^cKO^ mice presented reduced expression of the activation marker CD69 (Fig. 3g, h). Furthermore, we found that the number of IFNγ-producing inflammatory cells was also much lower in HFD-fed *B2m*^cKO^ mice than in control mice, with the greatest reduction observed in IFNγ^+^CD8^+^ T cells (Fig. 3i–k). Collectively, these findings suggest that ablation of B2M in adipocytes restricts the HFD-induced inflammatory response in EpiWAT, particularly by hindering the activation of CD8^+^ T cells and ATM polarization toward M1, thereby improving metabolic dysfunction. Therefore, in subsequent studies, we will focus on the mechanisms by which B2M in adipocytes is involved in CD8^+^ T-cell activation and M1 polarization.

### The activation of adipose-resident CD8^+^ T cells by hypertrophic adipocytes is dependent on B2M and direct cell contact

**Fig. 4:**
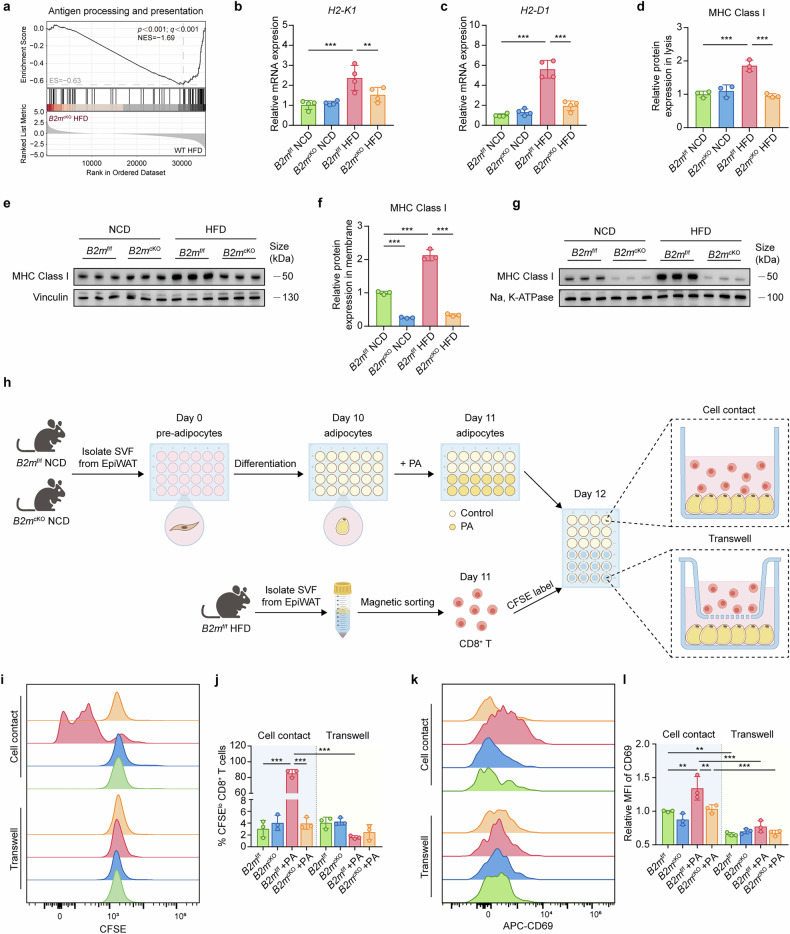
The activation of adipose-resident CD8^+^ T cell by hypertrophic adipocytes is dependent on B2M and direct cell contact. **a** GSEA of antigen processing and presentation pathway between HFD-fed WT mice and *B2m*^cKO^ mice. Relative mRNA levels of *H2-K1* (**b**) and *H2-D1* (**c**) in isolated adipocyte fraction of EpiWAT from NCD- or HFD-fed *B2m*^f/f^ and *B2m*^cKO^ mice (*n* = 4). Protein expressions and relative quantitation of MHC Class I in whole cell lysates (**d**, **e**) and membrane component (**f**, **g**) derived from adipocyte fraction isolated from EpiWAT of NCD- or HFD-fed *B2m*^f/f^ and *B2m*^cKO^ mice (*n* = 3). **h** Schematic representation of the in vitro co-culture experiment involving primary adipocytes and CD8^+^ T cells. CD8^+^ T cells were isolated from EpiWAT of obese *B2m*^f/f^ mice using a magnetic bead sorting kit and labeled with CFSE before co-culture. Adipocytes were induced from primary adipocyte precursors in SVF and treated with or without PA (2.5 mM) for 24 hours before co-culture. Flow cytometric analysis of proliferation (**i**, **j**) and CD69 (**k**, **l**) expression in CD8^+^ T cells following co-culture with primary adipocytes. (*n* = 3). The data are representative as the mean ± standard deviation (SD), with “n” representing the number of biological replicates per experimental group. Significant in **b**–**d**, and **f** was calculated using two-way ANOVA followed by Tukey’s HSD post hoc test for multiple comparisons. Significant in **j** and **l** was calculated using multi-way ANOVA followed by Tukey’s HSD post hoc test for multiple comparisons. *p < 0.05, **p < 0.01, ***p < 0.001. CFSE Carboxyfluorescein Succinimidyl Ester, EpiWAT epididymal adipose tissue, HFD high fat diet, NCD normal chow diet, PA palmitic acid, SVF stromal vascular fraction, WT wild type

Given the critical role of CD8^+^ T cells in adipose tissue inflammation and their reliance on MHC-I/B2M for activation,^16,24^ we hypothesized that hypertrophic adipocytes might participate in CD8^+^ T-cell activation through the MHC-I antigen presentation pathway during obesity. GSEA revealed that B2M ablation in adipocytes markedly suppressed the upregulation of the antigen processing and presentation pathway induced by HFD (Figs. 1b, 4a), indicating that hypertrophic adipocytes function as APCs capable of activating CD8^+^ T cells. We then examined the expression of MHC-I molecules in the EpiWAT-derived adipocytes of *B2m*^f/f^ or *B2m*^cKO^ mice fed an NCD or an HFD. A HFD significantly induced the upregulation of MHC-I mRNA and protein expression in adipocytes and, importantly, enhanced the localization of MHC-I on the cell membrane (Fig. 4b–g). These effects were abrogated by B2M deficiency, although under NCD conditions, B2M deficiency did not significantly affect MHC-I expression levels in adipocytes (Fig. 4b–g). These data suggest that an HFD markedly amplifies MHC-I antigen presentation in adipocytes, a response that is effectively blocked by B2M ablation.

Next, to determine whether hypertrophic adipocytes can directly activate adipose-resident CD8^+^ T cells in a B2M-dependent manner, we isolated SVFs from the EpiWAT of *B2m*^f/f^ or *B2m*^cKO^ mice, induced preadipocytes to differentiate into adipocytes, pretreated them with or without PA, cocultured these adipocytes with purified CD8^+^ T cells from EpiWAT via direct cell contact or separation via a transwell membrane (Fig. 4h). As expected, CD8^+^ T cells exhibited significant proliferation and activation exclusively when cocultured directly with PA-pretreated hypertrophic adipocytes from *B2m*^f/f^ mice, as demonstrated by increased cell division and upregulated CD69 expression (Fig. 4i–l). In contrast, adipocytes that were not pretreated with PA, subjected to B2M ablation, or separated in a transwell chamber failed to induce CD8^+^ T-cell activation and proliferation (Fig. 4i–l). Taken together, these data suggest that hypertrophic adipocytes act as APCs to potently activate adipose-resident CD8^+^ T cells in a B2M- and cell contact-dependent manner during obesity. Adipocyte-specific B2M deficiency effectively prevents CD8^+^ T-cell activation and expansion, thereby attenuating HFD-induced adipose inflammation.

### B2M deficiency prevents HFD-induced iron overload in adipocytes

**Fig. 5:**
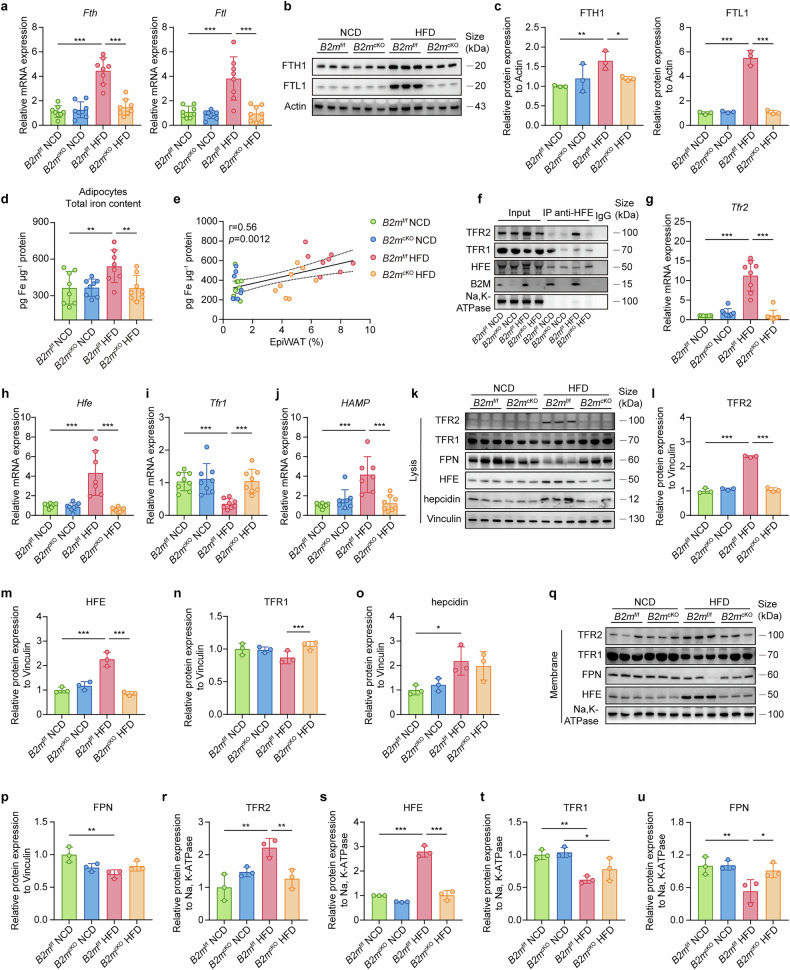
B2M deficiency prevents HFD-induced iron overload in adipocytes. **a** Relative mRNA levels of *Fth* and *Ftl* in the adipocyte fraction from EpiWAT of NCD- or HFD-fed *B2m*^f/f^ and *B2m*^cKO^ mice (*n* = 8). Protein expressions (**b**) and relative quantitation normalized to the internal reference Actin (**c**) of FTH1 and FTL1 in the lysates of mature adipocytes isolated from EpiWAT of NCD- or HFD-fed *B2m*^f/f^ and *B2m*^cKO^ mice (*n* = 3). **d** Total iron content, as measured by iron assay kit, in mature adipocytes from EpiWAT in NCD- or HFD-fed *B2m*^f/f^ and *B2m*^cKO^ mice (*n* = 8). **e** The correlation of total iron content in (**d**) with EpiWAT proportion (%) (*n* = 32). **f** Co-immunoprecipitation assay using HFE as bait protein showing the interaction between HFE, TFR1, and TFR2 within the membrane fractions of adipocytes isolated from EpiWAT in NCD- or HFD-fed *B2m*^f/f^ and *B2m*^cKO^ mice. Relative mRNA levels of *Tfr2* (**g**), *Hfe* (**h**), *Tfr1* (**i**), and *HAMP* (**j**) in the adipocyte fraction isolated from EpiWAT in NCD- or HFD-fed *B2m*^f/f^ and *B2m*^cKO^ mice (*n* = 7-8). Protein expressions (**k**) and relative quantitation normalized to the internal reference Vinculin in TFR2 (**l**), HFE (**m**), TFR1 (**n**), hepcidin (**o**), and FPN (**p**) in the lysates of adipocyte fraction isolated from EpiWAT in NCD- or HFD-fed *B2m*^f/f^ and *B2m*^cKO^ mice (*n* = 3). Protein expressions (**q**) and relative quantitation normalized to the internal reference Na,K-ATPase of TFR2 (**r**), HFE (**s**), TFR1 (**t**), and FPN (**u**) in membrane component derived from adipocyte fraction isolated from EpiWAT in NCD- or HFD-fed *B2m*^f/f^ and *B2m*^cKO^ mice (*n* = 3). The data are represented as the mean ± standard deviation (SD), with “n” representing the number of biological replicates per experimental group. Significant in **a**, **c**, **d**, **g**–**j**, **l**–**p**, and **r**–**u** was calculated using two-way ANOVA followed by Tukey’s HSD post hoc test for multiple comparisons. *p < 0.05, **p < 0.01, ***p < 0.001.EpiWAT epididymal adipose tissue, FTH ferritin heavy chain, FTL ferritin light chain, HFD high fat diet, HFE Hereditary hemochromatosis protein, NCD normal chow diet, TFR1 transferrin receptor 1, TFR2 transferrin receptor 2, FPN ferroportin

B2m is also involved in the iron uptake and transport pathway via interactions with hereditary hemochromatosis protein (HFE) and transferrin receptor (TFR), and this pathway is significantly upregulated in hypertrophic adipocytes under HFD stimulation (Fig. 1b, c). Notably, the DEGs in adipocytes from HFD-fed control and *B2m*^cKO^ mice were also enriched in pathways related to iron ion homeostasis (Supplementary Fig. 3d). To elucidate the role of B2M in iron homeostasis within adipocytes during obesity, we first assessed the iron load in adipocytes of *B2m*^f/f^ and *B2m*^cKO^ mice under NCD or HFD conditions. B2M knockout did not affect the iron homeostasis of adipocytes in lean NCD-fed mice. Compared with lean mice, HFD-fed *B2m*^f/f^ obese mice presented a significant increase in intracellular iron storage, as reflected by elevated mRNA and protein levels of ferritin heavy chain (FTH) and light chain (FTL), as well as increased total iron content in adipocytes, which was not observed in HFD-fed *B2m*^cKO^ mice (Fig. 5a–d). Moreover, the total iron level in adipocytes was positively correlated with the proportion of EpiWAT (Fig. 5e). These findings suggest that B2M plays a role in the iron storage response of adipocytes to HFD-induced obesity.

Given that HFE requires dimerization with B2M for stable membrane localization and then regulates iron uptake or hepcidin-ferroportin (FPN)-mediated iron efflux through switching interactions with TFR1 or TFR2, thereby contributing to hepatic and systemic iron homeostasis,^25,26^, we speculated that the HEF/B2M-TFR1/2-hepcidin-FPN axis may participate in the regulation of iron homeostasis in adipocytes. To this end, we conducted multiplex immunofluorescence staining on EpiWAT from NCD- or HFD-fed *B2m*^f/f^ and *B2m*^cKO^ mice and reported that HFE, TFR1, and TFR2 were all localized at the adipocyte membrane, with HFD-fed *B2m*^f/f^ mice exhibiting attenuated TFR1 and enhanced TFR2 expression on the adipocyte membrane compared with NCD-fed *B2m*^f/f^ mice. The colocalization coefficients indicated a preference for HFE to colocalize with TFR2 over TFR1 in hypertrophic adipocytes from HFD-fed *B2m*^f/f^ mice (Supplementary Fig. 5). However, under HFD conditions, B2M deficiency significantly impaired the expression of HFE on the cell membrane and decreased the colocalization of HFE with both TFR1 and TFR2 (Supplementary Fig. 5). Notably, coimmunoprecipitation assays confirmed a predominant HFE-TFR2 interaction on the membranes of adipocytes from obese mice and a more pronounced HFE-TFR1 interaction on the membranes of adipocytes from lean mice (Fig. 5f). Importantly, the absence of B2M disrupted HFE interactions with both TFR1 and TFR2 on the adipocyte membrane, regardless of dietary conditions (Fig. 5f). Furthermore, we observed an increase in TFR2 and HFE expression at both the mRNA and protein levels in the hypertrophic adipocytes of obese *B2m*^f/f^ mice fed a HFD compared with those of lean *B2m*^f/f^ mice, which was further validated by their expression of membrane protein components (Fig. 5g, h, k–m, and q–s). Conversely, the expression of *Tfr1* mRNA on the adipocyte membrane was decreased in hypertrophic adipocytes from obese *B2m*^f/f^ mice, although TFR1 remained unchanged in the cell lysate (Fig. 5i, k, n, q and t). Since the transfer of HFE from binding with TFR1 to TFR2 induces hepcidin expression^27^ and hepcidin impedes cellular iron efflux by negatively modulating the availability of the iron exporter FPN,^28^ we subsequently detected the expression of hepcidin and FPN in hypertrophic adipocytes. As expected, hepcidin mRNA and protein levels were increased in hypertrophic adipocytes of obese mice, accompanied by a corresponding decrease in the expression of FPN as well as its localization on the adipocyte membrane (Fig. 5j, k, o–q, t and u). However, these HFD-induced changes in adipocytes were abolished by adipocyte-specific B2M ablation (Fig. 5j, k, o–q, t and u). Collectively, these data suggest that the HFE/B2M-TFR1/2-hepcidin-FPN axis contributes to iron overload in adipocytes under HFD conditions. Moreover, B2M modulates iron homeostasis in adipocytes via the interaction between HFE and TFR1 and between HFE and TFR2.

Moreover, simultaneous detection of hepatic iron transport pathways revealed no significant changes in ferritin or iron transport-related proteins in the liver of 16-week-old HFD-fed mice compared with lean mice (Supplementary Fig. 6a, b). These findings indicate that under metabolic stress, the dysregulation of iron homeostasis in adipose tissue precedes that in the liver.

### B2M deficiency alleviates HFD-induced adipocyte ferroptosis and subsequent M1 polarization

**Fig. 6:**
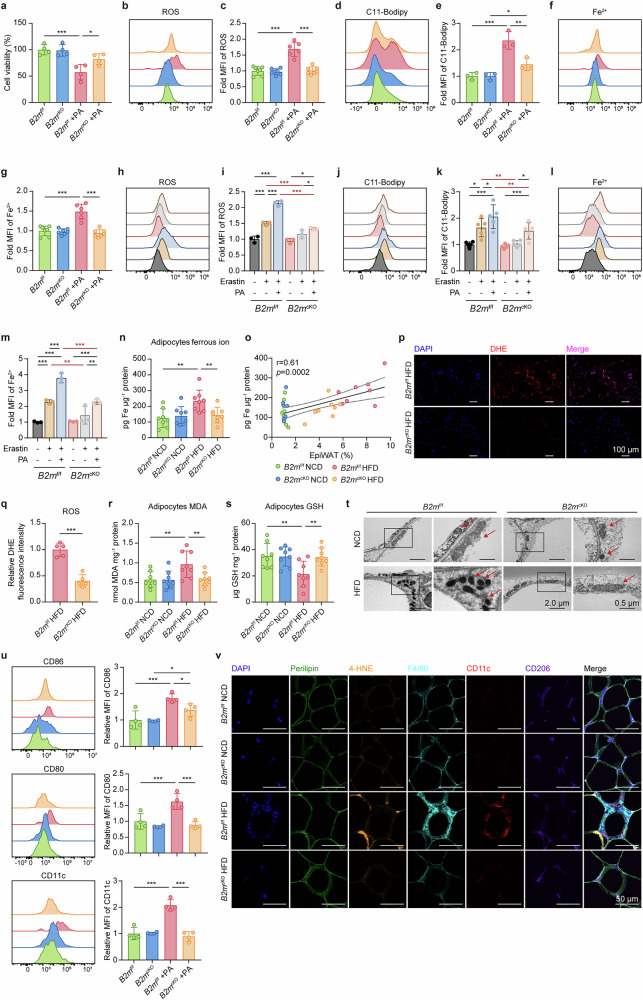
B2M deficiency alleviates HFD-induced adipocyte ferroptosis and subsequent M1 polarization. **a**–**g** Adipocytes were induced from primary adipocyte precursors in SVF of NCD-fed *B2m*^f/f^ and *B2m*^cKO^ mice and treated with or without PA (2.5 mM) for 24 hours. **a** cell viability (*n* = 4). The levels of ROS (**b**, **c**), lipid peroxides (**d**, **e**), and Fe^2+^ (**f**, **g**) in adipocytes were assessed using DCFH, C11-BODIPY^581/591^, and FeRhoNox-1 probes, respectively (*n* = 3-6). **h**–**m** Adipocytes were induced from primary adipocyte precursors in SVF from EpiWAT of NCD-fed *B2m*^f/f^ and *B2m*^cKO^ mice and treated with or without PA (2.5 mM) and/or Erastin (2 μM) for 24 hours. The levels of ROS (**h**, **i**), lipid peroxides (**j**, **k**), and Fe^2+^ (**l**, **m**) in adipocytes were assessed using DCFH, C11-BODIPY^581/591^, and FeRhoNox-1 probes, respectively (*n* = 3-5). **n** Ferrous ion content in mature adipocytes from NCD- or HFD-fed *B2m*^f/f^ and *B2m*^cKO^ mice, measured using an iron content detection kit (*n* = 8). **o** The correlation of ferrous ion content in (**n**) with EpiWAT proportion (%) in NCD- or HFD-fed *B2m*^f/f^ and *B2m*^cKO^ mice (*n* = 32). Representative images (**p**) and relative quantification (**q**) of ROS, visualized using staining fluorescent probe DHE, in EpiWAT of HFD-fed *B2m*^f/f^ and *B2m*^cKO^ mice, DAPI (blue). (*n* = 5). Scale bar, 100 μm. Levels of MDA (**r**) and GSH (**s**) in mature adipocytes isolated from EpiWAT in NCD- or HFD-fed *B2m*^f/f^ and *B2m*^cKO^ mice (*n* = 8). **t** Representative transmission electron microscopy images of the mitochondria (indicated by red arrow) of adipocytes in EpiWAT of NCD- or HFD-fed *B2m*^f/f^ and *B2m*^cKO^ mice. (*n* = 5-6). **u** Flow cytometry analysis of CD86, CD80, and CD11c expressions on ATMs following 24 hours of co-culture with primary adipocytes. ATMs were isolated from EpiWAT of lean *B2m*^f/f^ mice via F4/80^+^ magnetic beads. Primary adipocytes were differentiated from adipocyte precursors in SVF from EpiWAT of lean *B2m*^f/f^ or *B2m*^cKO^ mice and treated with or without PA (2.5 mM) for 24 hours prior to co-culture (*n* = 4). **v** Representative immunofluorescence staining images of ATMs in EpiWAT of NCD- or HFD-fed *B2m*^f/f^ and *B2m*^cKO^ mice, showing perilipin (green), 4-HNE (orange), F4/80 (cyan), CD206 (yellow), CD11c (red), and DAPI (blue). Scale bar, 50 μm. The data are represented as the mean ± standard deviation (SD), with “n” representing the number of biological replicates per experimental group. Significance in **q** was calculated using a two-tailed Student’s *t* test. Significant in **a**, **c**, **e**, **g**, **i**, **k**, **m**, **n**, **r**, **s**, and **u** was calculated using two-way ANOVA followed by Tukey’s HSD post hoc test for multiple comparisons. *p < 0.05, **p < 0.01, ***p < 0.001. 4-HNE 4-hydroxynonenal, ATMs adipose tissue macrophages, DHE Dihydroethidium, EpiWAT epididymal adipose tissue, GSH glutathione, HFD high fat diet, MDA malondialdehyde, NCD normal chow diet, PA palmitic acid, ROS reactive oxygen species, SVF stromal vascular fraction

Iron overload within cells may result in an iron-dependent Fenton reaction, which in turn produces toxic reactive oxygen species (ROS) and lipid peroxidation and ultimately induces ferroptosis.^29–31^ To explore whether ferroptosis occurs in hypertrophic adipocytes, we assessed the hallmarks of ferroptosis in hypertrophic 3T3-L1 adipocytes treated with PA in vitro. As expected, exposure to PA significantly increased the number of lipid droplets and decreased the viability of 3T3-L1 adipocytes (Supplementary Fig. 7a, b), along with elevated levels of free ferrous ions, ROS, and lipid peroxidation, all of which are indicative of ferroptosis (Supplementary Fig. 7c–e). However, the ferroptosis inhibitor ferrostatin-1 (Fer-1) effectively reversed these effects, indicating that ferroptosis occurs in hypertrophic adipocytes subjected to excessive nutritional stress in vitro (Supplementary Fig. 7b–e). In line with these findings, PA treatment significantly upregulated ferroptosis markers and induced cell death in primary adipocytes from *B2m*^f/f^ mice, whereas B2M knockout markedly attenuated these PA-induced effects (Fig. 6a–g). Notably, primary adipocytes from *B2m*^cKO^ mice—unlike those from *B2m*^f/f^ mice—exhibited significant resistance to ferroptosis induced by erastin (a ferroptosis inducer) alone or in combination with PA (Fig. 6h-m). These data suggest that B2M is indeed involved in the intrinsic regulation of ferroptosis in adipocytes.

Furthermore, obese mice fed a HFD exhibited a significant increase in the labile iron pool (LIP, free ferrous ion) in EpiWAT-derived adipocytes compared with lean mice fed an NCD, and the LIP content in adipocytes was positively correlated with the proportion of EpiWAT (Fig. 6n, o). Additionally, adipocytes from the EpiWAT of obese mice presented elevated levels of ROS and lipid peroxide-malondialdehyde (MDA) and decreased levels of the antiferroptotic metabolite glutathione (Fig. 6p–s). Notably, these ferroptotic characteristics were markedly ameliorated in the adipocytes of HFD-fed *B2m*^cKO^ mice (Fig. 6n–s). Transmission electron microscopy analysis further confirmed that a HFD resulted in mitochondrial atrophy and increased mitochondrial membrane density in hypertrophic adipocytes (Fig. 6t). However, the decrease in the mitochondrial morphology of adipocytes was reversed in the absence of B2M (Fig. 6t). Moreover, no obvious ferroptosis was detected in the livers of obese mice (Supplementary Fig. 7f), which is consistent with previous findings. These findings indicate that prior to an imbalance in liver iron homeostasis, HFD-induced iron overload in adipocytes can trigger ferroptosis, whereas the deletion of B2M in adipocytes can prevent this process.

To investigate whether hypertrophic adipocyte ferroptosis influences the capacity of adipocytes to activate adipose-resident CD8^+^ T cells, we pretreated primary adipocytes with or without PA and/or Fer-1, followed by direct coculture with CD8^+^ T cells sorted from EpiWAT. The results revealed that untreated or Fer-1-treated primary adipocytes failed to activate CD8^+^ T cells, whereas PA-pretreated adipocytes induced robust CD8^+^ T-cell activation and proliferation (Supplementary Fig. 7g, h). Notably, Fer-1 did not affect the ability of PA-pretreated adipocytes to activate CD8^+^ T cells (Supplementary Fig. 7g, h). These findings demonstrate that adipocyte ferroptosis is dispensable for activating adipose-resident CD8^+^ T cells. Furthermore, the results reinforce that B2M-mediated MHC class I antigen presentation and iron transport/ferroptosis pathways function independently, representing distinct yet parallel outcomes of metabolic stress.

Evidence has indicated that inflammatory ATMs (M1, CD11c^+^CDC206^-^) aggregate at the “crown-like” structure in obese adipose tissue, surrounding dead and dying adipocytes.^12^ On the basis of these findings, we propose that ferroptotic adipocytes may similarly be encircled by ATMs, potentially contributing to their polarization toward M1. To illustrate this, we cocultured 3T3-L1 adipocytes with RAW264.7 macrophages in vitro and found that hypertrophic 3T3-L1 adipocytes pretreated with PA induced M1 polarization in RAW264.7 cells, as evidenced by upregulated expression of M1 markers (CD86, CD80, CD11c, and *iNOS*) and proinflammatory cytokines (*Tnfa*, *Il1b*, and *Il6*), which was effectively abrogated by Fer-1 pretreatment (Supplementary Fig. 7i–l). Consistently, coculture experiments involving primary adipocytes and ATMs from EpiWAT (Supplementary Fig. 7m) revealed that adipocytes from *B2m*^f/f^ mice pretreated with PA promoted M1 polarization in ATMs, whereas Fer-1 effectively blocked this effect, suggesting that hypertrophic adipocytes indeed promote M1 polarization via ferroptosis. Conversely, primary adipocytes that were either not pretreated with PA or derived from *B2m*^cKO^ mice, regardless of PA treatment, failed to induce M1 polarization in ATMs (Fig. 6u). Additionally, multiplex immunofluorescence staining of EpiWAT revealed that, compared with those from lean mice, EpiWAT from obese mice presented clear signs of ferroptotic adipocytes (marked by the lipid peroxidation product 4-HNE), accompanied by a significant increase in F4/80^+^ ATMs, predominantly CD11c^+^ M1 ATMs, encircling adipocytes and forming crown-like structures (Fig. 6v). In contrast, EpiWAT from HFD-fed *B2m*^cKO^ mice presented immunostaining characteristics similar to those of lean mice (Fig. 6v). Collectively, these findings suggest that a HFD induces ferroptosis in adipocytes, subsequently triggering the M1 polarization of adjacent ATMs and aggravating adipose inflammation, whereas B2M ablation ameliorates adipocyte ferroptosis and limits the amplification of the M1-type inflammatory response in adipose tissue.

TUNEL staining results revealed that B2M ablation significantly attenuated adipocyte death in the EpiWAT of HFD-fed mice (Supplementary Fig. 7n, o). We further questioned whether B2M deletion, in addition to its role in protecting adipocytes against ferroptosis, exerts additional effects on other types of cell death. Previous studies have shown that in obesity, adipocyte apoptosis and adipose tissue inflammation reinforce each other in a vicious cycle, with apoptotic adipocytes contributing to crown-like structure (CLS) formation and promoting M1 macrophage polarization. To investigate the effects of B2M knockout on energy excess-induced adipocyte apoptosis and ferroptosis without the indirect influence of the microenvironment of adipose tissue in vivo, we first validated that PI/Annexin-V double staining can distinguish the two death types via 3T3-L1 adipocytes treated with the apoptosis inducer H₂O₂ or the ferroptosis inducer erastin: apoptosis was characterized by Annexin-V^+^/PI^+/-^, and ferroptosis was characterized by PI^+^Annexin-V^-^ (Supplementary Fig. 7p–r). Then, we differentiated adipocyte precursor cells from *B2m*^f/f^ and *B2m*^cKO^ mice into mature adipocytes in vitro and subsequently treated them with PA, followed by flow cytometric analysis via PI/Annexin-V staining. The results demonstrated that B2M deficiency significantly reduced only the proportion of PI⁺Annexin-V⁻ cells, with no obvious change in the Annexin-V⁺ population (Supplementary Fig. 7s–u). These findings suggest that B2M knockout selectively inhibits adipocyte ferroptosis—rather than apoptosis—via a cell-intrinsic mechanism under energy excess stress.

### Therapeutic attenuation of obesity and metabolic disorders by EpiWAT-specific B2M knockdown

**Fig. 7:**
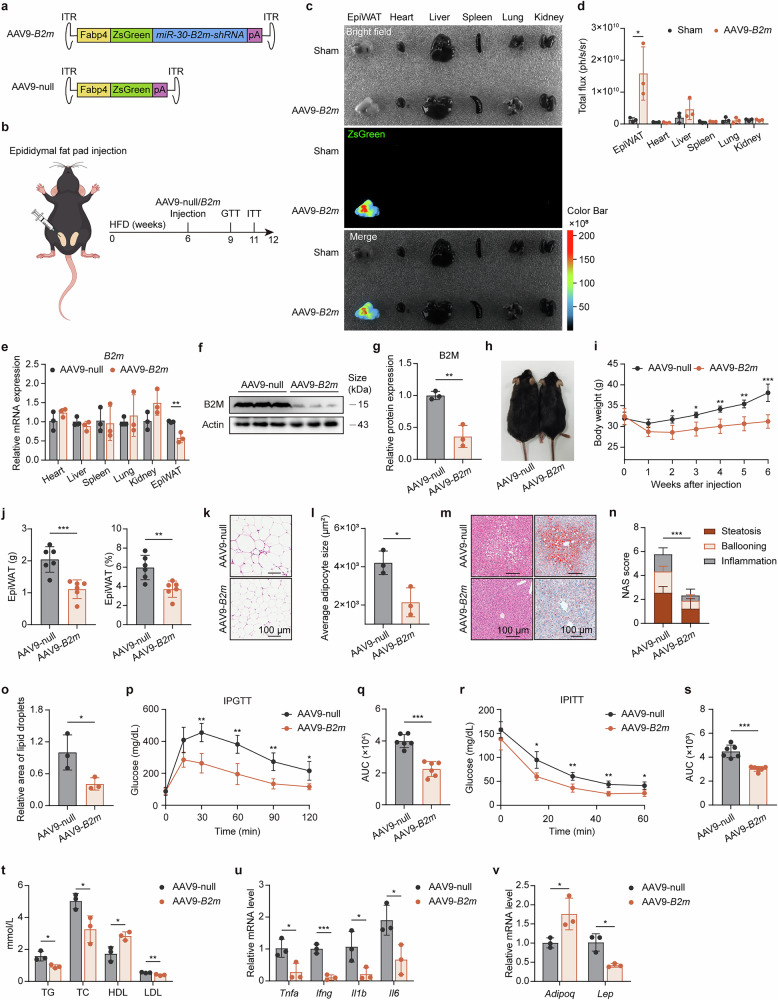
AAV-mediated B2M Knockdown in EpiWAT Ameliorates Obesity and Metabolic Disorders. **a** Overview for AAV9-mediated knockdown of B2M in EpiWAT. **b** Experiment scheme of male mice was injected with either AAV9-Fabp5-ZsGreen-*B2m-*shRNA (AAV9-*B2m*) or AAV9-Fabp5-ZsGreen (AAV9-null) following six-week HFD feeding and subsequently maintained on the same diet for an additional 6 weeks. **c** Ex vivo organ fluorescence imaging of EpiWAT, Heart, Liver, Spleen, Lung and Kidney from AAV9-*B2m* mice and age-matched sham-operated mice at 2 weeks post-injection. **d** Fluorescence intensity of tissues in **c** (*n* = 3). **e** Relative *B2m* mRNA expression in EpiWAT, Heart, Liver, Spleen, Lung and Kidney (*n* = 3). Protein expression (**f**) and relative quantification (**g**) of B2M in adipocytes derived from EpiWAT (*n* = 3). **h** Representative photograph of AAV9-null and AAV9-*B2m* mice. **i** Body weights (*n* = 6). **j** Weight and proportion of EpiWAT (*n* = 6). Representative histological images showing H&E staining of EpiWAT (**k**), with quantitation of average adipocyte size (**l**) (*n* = 3). Scale bar, 100 μm. **m**–**o** Representative histological images showing H&E staining and Oil Red O staining of liver sections, with NAS (**n**) and relative quantitation of Oil Red O staining (**o**) (*n* = 3). Scale bar, 100 μm. **p**–**s** IPGTT (2 g glucose per kg body weight), IPITT (0.75 U insulin per kg body weight) and their respective AUC (*n* = 6). **t** Serum levels of TG, TC, HDL and LDL (*n* = 3). Relative mRNA levels of inflammatory cytokines (**u**) and adipocytokines (**v**) in EpiWAT (*n* = 3). The data are representative as the mean ± standard deviation (SD), with “n” representing the number of biological replicates per experimental group. Significant in **d**, **e**, **g**, **j**, **l**, **o**, **q**, and **s**–**v** was calculated using a two-tailed Student’s *t* test. Significant in **i,** **p** and **r** was calculated using two-way ANOVA followed by Tukey’s HSD post hoc test for multiple comparisons. Significant in **n** was calculated using Mann-Whitney *U* test. *p < 0.05, **p < 0.01, ***p < 0.001. AAV9 type 9 adeno-associated virus, AUC area under the curve, EpiWAT epididymal adipose tissue, HDL high density lipoprotein, HFD high-fat diet, IPGTT intraperitoneal glucose tolerance test, IPITT intraperitoneal insulin tolerance test, LDL low density lipoprotein, NAS Non-alcoholic fatty liver disease activity score, Sham sham-operated group, TC cholesterol, TG triglyceride

To further investigate the therapeutic potential of specific interventions for B2M expression in adipose tissue during obesity, we employed an adipose-targeted adeno-associated virus type 9 (AAV9) vector carrying *B2m*-shRNA (AAV9-*B2m*) or ZsGreen (AAV9-null control) (Fig. 7a, b). Ex vivo fluorescence imaging validated the adipose-targeting specificity of the AAV9 vectors (Fig. 7c, d). In the mice treated with AAV9-*B2m*, the expression of *B2m* was significantly suppressed in the EpiWAT, whereas its expression in other major organs remained unaffected (Fig. 7e–g). Compared with AAV9-null controls, AAV9-*B2m* intervention effectively prevented HFD-induced body weight gain, adipose accumulation, and adipocyte hypertrophy (Fig. 7h–l). Additionally, this intervention reduced ectopic lipid deposition in the liver and reversed HFD-induced glucose intolerance and insulin resistance in mice (Fig. 7m–s). Furthermore, AAV9-*B2m* intervention ameliorated dyslipidemia and adipokine abnormalities while also reducing the transcriptional levels of inflammatory factors in EpiWAT (Fig. 7t–v). Collectively, these findings demonstrate that interfering with B2M expression in EpiWAT during obesity progression effectively alleviates obesity and associated metabolic disorders.

### *B2m* expression in adipose tissue is associated with human obesity

**Fig. 8:**
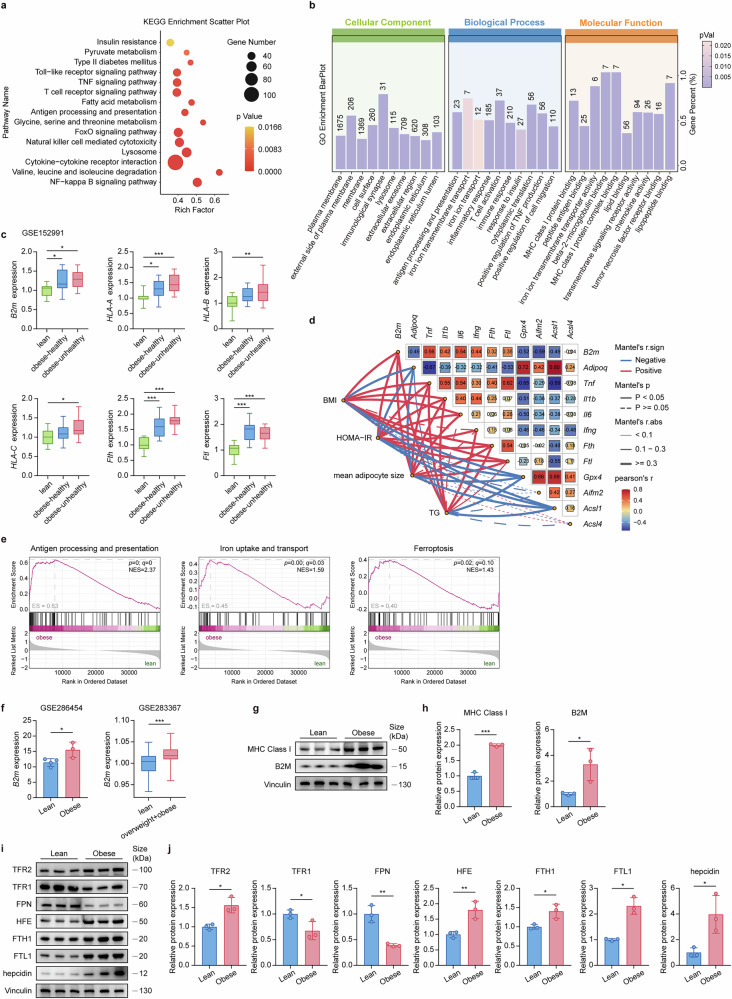
B2M expression in adipose tissue is associated with human obesity. KEGG bubble diagram (**a**) and GO bar chart (**b**) displaying selected enrichment pathways of DEGs in SAT from lean and metabolically unhealthy obese patients. **c** Relative expression of *B2m*, *HLA-A*, *HLA-B*, *HLA-C*, *Fth* and *Ftl* gene in SAT across lean patients (*n* = 11), metabolically healthy obese patients (*n* = 14) and metabolically unhealthy obese patients (*n* = 20). **d** Correlation analysis of indicated genes and the Mantel test analysis of clinical indicators (BMI, HOMA-IR and mean adipocyte size) and indicated genes among lean and obese-unhealthy patients. A color gradient is utilized to denote Person’s correlation with the value reflecting the precise value of this coefficient. The color of the line indicates the correlation direction in the Mantel test (Mantel’s r. sign), with red indicating a positive correlation and blue signifying a negative correlation. The thickness of the lines reflects the strength of the correlation in the Mantel test (Mantel’s r.abs): thin lines for weak (r < 0.1), standard lines for moderate (0.1 < r < 0.3), and thick lines for strong (r >= 0.3) correlations. The type of line denotes the significance of the Mantel test (Mantel’s p), with solid indicating a significant correlation and dashed signifying a non-significant correlation. **e** GSEA of DEGs showing significant upregulation of three pathways in SAT from lean patients and metabolically unhealthy obese patients. **f** Relative expression of *B2m* in VAT. GSE286454 dataset with lean (n = 4) and obese (n = 3) and GSE283367) with lean (n = 70) and obese (n = 43). **g**–**j** Protein expressions and relative quantification of B2M, MHC Class I, TFR2, TFR1, FPN, HFE, FTH, FTL and hepcidin in whole cell lysates derived from adipocyte fraction isolated from VAT across lean patients and obese patients (*n* = 3). The data are representative as the mean ± standard deviation (SD), with “n” representing the number of biological replicates per experimental group. Significant in **f**, **h**, and **j** was calculated using a two-tailed Student’s *t* test. Significant in **c** was calculated using one-way ANOVA followed by Tukey’s HSD post hoc test for multiple comparisons. *p < 0.05, **p < 0.01, ***p < 0.001. B2M β2-microglobulin, BMI body mass index, DEGs differentially expressed genes, FTH ferritin heavy chain, FTL ferritin light chain, HFE Hereditary hemochromatosis protein, HOMA-IR homeostasis model assessment of insulin resistance, SAT subcutaneous adipose tissue, TFR1 transferrin receptor 1, TFR2 transferrin receptor 2, FPN ferroportin, VAT visceral adipose tissue

To determine the relevance of B2M in human obesity, we analyzed RNA-seq data (GEO: GSE152991) of SAT from both obese individuals and healthy lean controls. KEGG and GO enrichment analyses of DEGs related to SAT between lean controls and unhealthy obese individuals revealed significant enrichment in genes related to antigen processing and presentation, as well as iron ion transport (Fig. 8a, b). Notably, there was significant upregulation of *B2m*, *HLA-ABC*, *Fth* and *Ftl* expression in the SAT of obese individuals compared with controls, with the highest expression level observed in those classified as obese-unhealthy individuals, except for *Ftl* (Fig. 8c). Importantly, multiple correlation analysis (Fig. 8d) revealed that *B2m* expression in SAT was positively associated with BMI, homeostatic model assessment of insulin resistance (HOMA-IR), adipocyte size, and triglyceride (TG) levels, as were the expression levels of inflammatory cytokine genes (*Tnf*, *Il1b*, *Il6*, and *Ifng*) and iron storage-related genes (*Fth* and *Ftl*). Conversely, *B2m* expression in SAT was negatively correlated with adiponectin (*Adipoq*) and antiferroptotic genes (*Gpx4* and *Aifm2*). Furthermore, GSEA revealed that in patients with obesity, the antigen processing and presentation, iron uptake and transport, and ferroptosis pathways associated with *B2m* were significantly upregulated (Fig. 8e). To further validate these findings, analysis of two other independent RNA-seq datasets (GEO: GSE286454 and GSE283367) of human visceral adipose tissue (VAT) revealed that *B2m* expression was significantly greater in the VAT of patients with obesity than in that of healthy controls (Fig. 8f). To strengthen the translational relevance of B2M, we measured the expression of B2M and related molecules in human adipocyte samples. Compared with those from lean controls, adipocytes from VAT from obese patients presented significant upregulation of B2M and MHC-I, as well as iron transport and storage-related proteins (Fig. 8g–j). This finding in patients with obesity aligns with the conclusions drawn from the aforementioned obese mice. Collectively, these findings suggest that the aberrant increase in B2M in human adipose tissue is linked to adipose tissue inflammation and iron excess, both of which are implicated in human obesity-related metabolic disorders.

## Discussion

**Fig. 9:**
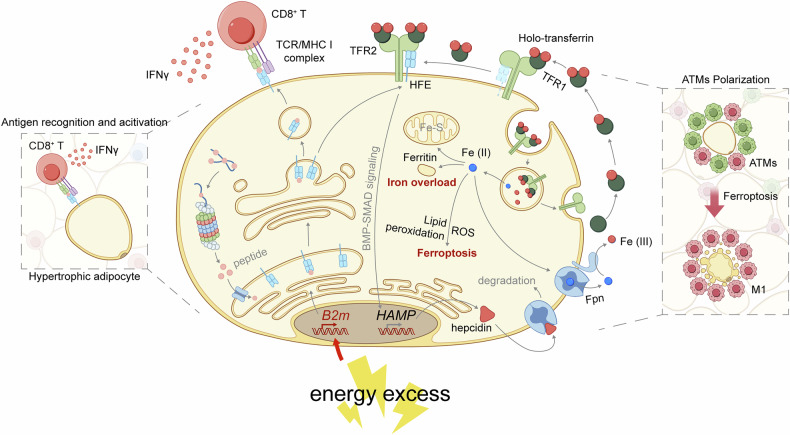
Summary scheme. A schematic illustration of the proposed mechanism by which hypertrophic adipocytes induce chronic adipose inflammation and metabolic disorders associated with obesity. Excessive energy stimulates adipocytes to upregulate B2M expression, which not only facilitates the activation and proliferation of CD8^+^ T cells in adipose tissue through the endogenous antigen presenting pathway, but also promotes iron overload and subsequent ferroptosis of hypertrophic adipocytes via the HFE/B2M-TFR2-Hepcidin-FPN axis, leading to the polarization of ATMs towards M1, thereby accelerating chronic inflammation metabolic disorders. ATMs, adipose tissue macrophages. This figure was created using Adobe Illustrator

Our study demonstrated the pivotal role of adipocytes in driving chronic inflammation and metabolic disorders in obesity via two B2M-dependent yet mechanistically distinct pathways. First, hypertrophic adipocytes potently activated adipose-resident CD8^+^ T cells in a B2M- and cell contact-dependent manner. Concurrently, hypertrophic adipocytes undergo iron overload and ferroptosis via the HFE/B2M-TFR2-hepcidin-FPN axis, thereby promoting M1 polarization of ATMs (Fig. 9).

Emerging evidence suggests that adipose-resident CD8^+^ T-cell activation is antigen driven.^32–34^ However, whether adipocytes act as APCs to activate adipose-resident CD8^+^ T cells remains to be determined. Our RNA-seq data revealed significant upregulation of the MHC-I antigen presentation pathway in hypertrophic adipocytes, which was further evidenced by elevated expression levels of B2M and MHC-I molecules, particularly in the membrane protein fractions. Crucially, coculture experiments demonstrated that hypertrophic adipocytes were capable of directly activating adipose-resident CD8^+^ T cells in a B2M- and direct cell contact-dependent manner. Consistently, the knockout of B2M in adipocytes effectively alleviated HFD-induced accumulation and activation of CD8^+^ T cells within adipose tissue. Together, our observations strongly suggest that hypertrophic adipocytes can provide endogenous antigen signals to mediate adipose-resident CD8^+^ T-cell activation and proliferation, thereby contributing to adipose inflammation. Future research should focus on identifying obesity-associated endogenous antigens presented by MHC-I molecules on the surface of hypertrophic adipocytes to elucidate the mechanisms underlying obesity-related adipose inflammation.

We also found that adipocyte-specific B2M knockout reduced the HFD-induced aggregation of iNKT cells in adipose tissue. Since iNKT cells are activated upon recognizing glycolipid antigens presented by CD1d, which forms a stable complex with B2M on APC surfaces, we propose that hypertrophic adipocytes might also provide antigenic signals for adjacent iNKT cell activation through CD1d/B2M. Although direct evidence is lacking here, our hypothesis supports Jin Young Huh et al.‘s findings that hypertrophic adipocytes upregulate CD1d, engaging in adipose-resident iNKT cell activation, and that CD1d knockout in adipocytes reduces these cells in adipose tissue.^35^

The HFE/B2M-TFR2-hepcidin-FPN axis serves as a core pathway regulating iron homeostasis, ensuring precise iron delivery to iron-requiring proteins while limiting potential toxicity, such as iron-dependent oxidative damage.^26,28^ Iron ions (Fe³⁺) combine with transferrin (TF) to form TF-Fe³⁺ complexes (Holo-transferrin), which then enter cells via TFR1 on the cell surface. HFE/B2M regulates cellular iron influx by competing with Holo-transferrin for binding to TFR1.^28^ Under high-iron conditions, Holo-transferrin outcompeted HFE/B2M, thereby facilitating the dissociation of HFE/B2M from TFR1 and its subsequent binding to TFR2. The formation of the HFE/B2M-TFR2 complex activates downstream SMAD signaling, ultimately inducing the expression of the iron-regulatory hormone hepcidin (encoded by the H*AMP* gene).^27,28^ Hepcidin decreases iron efflux by promoting the internalization and degradation of FPN and/or directly obstructing the iron channel, thereby inhibiting iron release and increasing the intracellular iron content.^28^ Although previous studies have elucidated the mechanism through which the HFE/B2M-TFR2-hepcidin-FPN axis regulates hepatic iron homeostasis,^27,28^ its involvement in obesity-related adipocyte iron overload is not yet fully understood.

Evidence from mice and humans, including our analysis of human transcriptome data, consistently indicates iron overload in the adipose tissue of obese individuals.^36–39^ Recent research by Zhang et al. further demonstrated that a HFD induces iron overload in adipocytes and that adipocyte-specific TFR1 knockout or FPN overexpression can reduce the iron content in adipocytes, thereby alleviating obesity and metabolic disorders in HFD-fed mice.^36^ However, the underlying mechanisms of HFD-induced iron overload in adipocytes remain to be fully elucidated. Our study revealed for the first time that both HFE and TFR2 are markedly upregulated in hypertrophic adipocytes, accompanied by increased colocalization, whereas HFE mainly binds to TFR1 in steady-state adipocytes. The formation of the HFE-TFR2 complex promotes downstream hepcidin expression and decreases membrane-localized FPN, ultimately leading to impaired iron efflux and intracellular iron overload in hypertrophic adipocytes. Although this mechanism resembles the previously reported hepatic iron metabolism pathway, our findings demonstrate that 16-week HFD challenge is sufficient to ina duce significant changes in the HFE/B2M-TFR2-hepcidin-FPN axis and iron overload in adipocytes without altering this axis or causing notable iron deposition in the liver. Moreover, previous studies have reported that the liver requires more than 20 weeks of HFD intervention to exhibit notable iron deposition.^29,40^ These findings suggest that adipocytes may exhibit greater sensitivity to iron metabolism dysregulation under energy excess stress. In this study, B2M knockout destabilized HFE on the adipocyte membrane, disrupting the HFE/B2M-TFR2-hepcidin-FPN axis and preventing HFD-induced iron overload. Nevertheless, studies by Erica Martins, Nolwenn Joffin et al. did not observe significant changes in TFR1 and hepcidin mRNA levels in hypertrophic adipocytes, potentially because of the different mouse models utilized or HFD conditions used.^39,41^

Ferroptosis is an iron-dependent cell death mechanism characterized by iron overload, ROS and lipid peroxidation.^30^ Ferroptotic, cells release various damage-associated molecular patterns (DAMPs), including ATP, high-mobility group box 1 (HMGB1), heat shock proteins (HSP70 and HSP90), and polyunsaturated fatty acids (PUFAs), which are responsible for macrophage recruitment and M1 polarization.^42–45^ Our data demonstrated that hypertrophic adipocytes from obese mice presented typical ferroptotic features, which are absent in homeostatic adipocytes from lean mice. Although hepatic ferroptosis has been well characterized in the pathogenesis of MAFLD,^29,40^ our data show that, under HFD conditions, the upregulation of ferroptosis markers (such as MDA and ferrous ions) in adipocytes precedes that in the liver, indicating that adipocytes exhibit greater susceptibility to ferroptosis. This view is also supported by our finding that obesity-induced perturbations in the HFE/B2M-TFR2-hepcidin-FPN axis and iron overload occur earlier in adipocytes than in the liver.

Importantly, coculture experiments revealed that hypertrophic adipocytes with ferroptotic characteristics effectively promoted M1 polarization, a phenomenon that was eliminated when these adipocytes were cocultured with normal or B2M-deficient adipocytes or when a ferroptosis inhibitor was used. Consistently, B2M ablation attenuated HFD-induced adipocyte ferroptosis, accompanied by a significant decrease in ATM accumulation in EpiWAT, particularly in the M1 subtype. This phenomenon was also verified at the histological level. Therefore, we propose that an HFD induces iron overload and ferroptosis in hypertrophic adipocytes through B2M-involved iron transport mechanisms, potentially releasing certain DAMPs that induce M1 polarization, exacerbating adipose inflammation and metabolic disorders. However, contrary to our findings, a recent study highlighted the beneficial role of ferroptosis signaling in adipocytes in combating obesity.^46^ These findings indicate that activating ferroptosis signaling in adipocytes can trigger thermogenic programs, thereby reducing lipid accumulation and preventing HFD-induced obesity and metabolic disorders. We believe that several factors may explain these discrepancies. First, Wang et al. focused on the thermogenic capacity of adipocytes, whereas we focused on adipose tissue inflammation. Second, Wang et al. characterized ferroptosis in whole adipose tissue rather than primary adipocytes by measuring lipid peroxidation and nonheme-bound iron rather than free iron ions, suggesting that ferroptosis signaling is significantly weakened in hypertrophic adipose tissue. In contrast, we used primary adipocytes to measure the levels of ROS, lipid peroxidation, and nonheme-bound iron as well as free iron ions, which demonstrated that hypertrophic adipocytes exhibit significantly increased ferroptosis. Finally, there were significant differences in the RNA-seq samples and the mouse models used in the two studies. Nonetheless, we acknowledge the possibility that ferroptosis signaling in adipocytes may modulate thermogenesis and proinflammatory responses through distinct mechanisms, thereby playing different roles in obesity.

In obese animals and humans, more than 90% of the macrophages in adipose tissue aggregate around dead adipocytes, a characteristic morphology known as CLS.^47,48^ These macrophages exhibit an M1 proinflammatory phenotype and are actively involved in initiating and promoting adipose tissue inflammation.^49,50^ Beyond adipocyte ferroptosis—the focus of our study—emerging evidence suggests that adipocytes may undergo apoptosis during the process of hypertrophy due to factors such as hypoxia or endoplasmic reticulum stress.^51,52^ Consequently, these apoptotic adipocytes contribute to the formation of CLSs and promote the polarization of macrophages toward the M1 phenotype.^53^ Our in vitro experiments demonstrated that under energy excess stress, B2M knockout may selectively suppress adipocyte ferroptosis via a cell-intrinsic mechanism without affecting apoptosis. However, the possibility that B2M knockout indirectly protects against adipocyte apoptosis through immune regulation cannot be ruled out, for example, by interfering with MHC class I-restricted CD8⁺ T-cell recognition and the induction of adipocyte apoptosis or by reducing the levels of proinflammatory cytokines in adipose tissue, such as TNF-α and IL-6, thereby indirectly mitigating adipocyte apoptosis. Notably, B2M knockout fails to completely abolish adipocyte death under HFD challenge. This is likely attributed to the presence of other B2M-independent cell death mechanisms—such as the pathway triggered by “lipotoxicity-oxidative stress-microenvironment damage”—which ultimately results in the “residual dead adipocytes” observed in the VAT of B2m^cKO^ mice. Therefore, subsequent studies need to further explore the primary type of adipocyte death in obesity, as well as its relative contributions to macrophage infiltration, M1 polarization and chronic adipose tissue inflammat,ion.

Furthermore, AAV9-mediated targeted reduction in B2M in adipose tissue effectively ameliorated HFD-induced obesity, adipose inflammation and metabolic abnormalities in mice,. These findings provide a key foundation for the development of highly efficient, specific adipose B2M inhibitors to treat obesity and related chronic inflammation and metabolic disorders. More importantly, our analysis of transcriptome data from human SAT and VAT, combined with B2M detection in human adipocyte samples, consistently confirms the significant role and clinical translational value of B2M in obesity-related chronic inflammation and metabolic dysfunction in humans.

In conclusion, our study reveals that hypertrophic adipocytes engage in B2M-dependent mechanisms concurrently to mediate the inflammatory activation of both adipose-resident CD8^+^ T cells and ATMs. Additionally, our findings highlight the translational potential of targeting adipocyte B2M inhibition as a therapeutic strategy for obesity-related chronic inflammation and metabolic disorders.

## Materials and methods

### Mouse models

The animal experiments were approved by the Institutional Animal Care and Use Committees of the Army Medical University (no. AMUWEC20218024).

#### Cre-Loxp

To establish a mouse model with adipocyte-specific B2M knockout, *B2m*^f/f^ mice were crossed with *Adipoq*-Cre mice. CRISPR-Cas9 technology was utilized to generate *B2m*^f/f^ mice, in which loxP sequences were inserted into intron 1 and intron 3 of the *B2m* gene in C57BL/6J mice. These wild-type C57BL/6J mice were bred, followed by PCR genotyping and sequencing of the offspring. For 3’ loxP insertion genotyping, the primers used were 5’- ATTCTTTTATCCTGTGGACTGGCA-3’ and 5’- CTGTACAAAGACAGCAAGCTCA-3’. The wild-type band measured 226 bp, and the loxP band was 348 bp. *Adipoq*-Cre mice (C001042) were purchased from Cyagen.

#### Mouse breeding

During the experimental period, all the mice were housed in an SPF-grade animal facility with the temperature strictly maintained at 22–24 °C, the relative humidity controlled at 40–60%, and a 12-h light/dark cycle implemented, fully compliant with the environmental parameter requirements for SPF-level animal facilities outlined in the *Guidelines for the Care and Management of Laboratory Animals*. The mice had ad libitum access to food and water.

#### Diet-induced obesity model

Four-week-old C57BL/6J mice were randomly assigned to either a high-fat diet (HFD) group or a normal control diet (NCD) group. *B2m*^cKO^ and *B2m*^f/f^ were also grouped in the same pattern. For the HFD studies, the mice were fed a diet with 60% calories from fat (no. D12492, Research Diets) for 12–16 weeks to induce obesity, whereas the mice in the NCD group were fed standard rodent feed with 10% calories from fat (no. D12450B, Research Diets). The body weights of the groups of mice were measured weekly at a fixed time. Daily food intake was calculated by dividing the net feed consumption per day by the number of mice per group.

### Human specimens

This study protocol was approved by the Clinical Research Ethics Committee of the Southwest Hospital of Army Medical University (Approval No. (A) KY2025062). All the subjects provided written informed consent prior to participation. Visceral adipose tissue samples were collected from 6 patients (aged 18–70 years, with a BMI of 20–25, not including 25, in the lean group and ≥25 in the obese group) who underwent abdominal surgery at Southwest Hospital of Army Medical University (see Supplementary Table 1). The exclusion criteria included fever or infection, renal disease, organ transplantation, steroid use, estrogen replacement, AIDS, >10% body weight change within the prior 3 months, or previous diagnosis of type 1 diabetes, hemochromatosis, or lipodystrophy. Immediately after sampling, the tissues were transferred to ice-cold saline and rapidly processed for adipocyte isolation.

### Cell lines

3T3-L1 and RAW264.7 cells were maintained in DMEM containing 10% fetal bovine serum (FBS) and 1% penicillin/streptomycin in a humidified incubator with 5% CO_2_ at 37 °C.

Administration of AAV vectors

#### Construction and Injection of AAV

AAV was administered as previously described.^54^ AAV serotype 9, driven by the Fabp4 promoter, was purchased from HanBio Technology (Shanghai, China). AAV-ZsGreen-miR30-shRNA (*B2m*) (AAV9-*B2m*) was used to knockdown B2M in EpiWAT, while AAV-ZsGreen (AAV-null) was used as the control. The *B2m*-shRNA sequence was 5’ GCAGAGUUAAGCAUGCCAGUA 3’. AAV-*B2m*-shRNA or AAV-ZsGreen was administered via direct injection into the bilateral EpiWAT. For delivery of AAV to the EpiWAT, the mice were anesthetized with ketamine (100 mg/kg) and xylazine (10 mg/kg), followed by laparotomy to expose the EpiWAT. Subsequently, 50 μL of AAV solution was administered at multiple sites within each fat pad, and 50 μL of AAV solution was injected into each fat pad at multiple spots (5–8 spots per fat pad), with a total viral titer of 1 × 10^12^ viral genomes, to ensure widespread vector distribution within the EpiWAT. Finally, the surgical incision was closed with sutures.

#### Ex vivo organ fluorescence imaging

Two weeks after the administration of AAV9-*B2m*, the heart, lung, kidney, spleen, liver, and EpiWAT from AAV9-*B2m*-injected mice and age-matched sham-operated mice were rapidly isolated. The organs were rinsed three times with precooled PBS, and excess surface liquid was carefully removed before the tissues were arranged in an organized manner. Ex vivo fluorescence imaging was conducted via an in vivo imaging system (IVIS Spectrum, PerkinElmer) equipped with an excitation wavelength of 488 nm and an emission filter range of 503–563 nm, with an exposure time of 0.5 seconds. The fluorescence signal intensity was quantified via Kuant software.

### Body composition analysis and microcomputed tomography (micro-CT)

Fat, lean and water masses were measured via a nuclear magnetic resonance (NMR) spectrometer (Niumag Analytical Instrument Corporation). Imaging of the subcutaneous and visceral fat in the trunk was conducted via micro-CT (SkyScan 1276, Bruker). Grayscale image slices were reconstructed into 3D tomograms via NRecon software (V1.7.4.2, Bruker).

### Isolation of mature adipocytes and stromal-vascular fraction (SVF)

SVFs and mature adipocytes were isolated as previously described.^55^ Briefly, EpiWAT from male mice was weighed, rinsed in PBS, minced and subsequently digested with DPBS without Ca^2+^ or Mg^2+^ supplemented with 0.5% BSA (SIGMA, V900933) and 2 mg ml^−1^ collagenase II (SIGMA, C6885) for 30 min at 37 °C in a shaker at 200 rpm. DPBS (containing 0.5% BSA) was added to terminate the digestion. The sample was then centrifuged at 350 × *g* for 5 min. Floating mature adipocytes were collected for further measurements. The remaining pellet containing the SVF was resuspended in RBC lysis buffer, followed by the addition of cold PBS to stop the lysis process. The cell suspension was filtered through a 70 μm cell strainer and centrifuged again at 350 × *g* for 5 min at 4 °C to obtain the cell pellet.

The cell pellets were either used for in vitro culture or subjected to flow cytometry analysis.

After the remaining supernatant was discarded, the pelleted cells were resuspended in red blood cell lysis buffer. Lysis was stopped by the addition of cold PBS (containing 2% FBS), followed by filtration through a 70 μm cell strainer and centrifugation for 5 min at 350 × *g* and 4 °C to pellet the cells. The pelleted cells were either cultured or analyzed via flow cytometry as described below.

### Primary preadipocyte and 3T3-L1 cell differentiation

#### Primary preadipocyte differentiation

Fresh SVFs isolated from EpiWAT were cultured in growth medium (DMEM containing 10% FBS and 1% penicillin‒streptomycin) until they reached confluence and then induced with an adipogenic differentiation cocktail consisting of 1 μM dexamethasone (Selleck, S1322), 0.5 mM isobutylmethylxanthine (MCE, HY-12318), 5 μg/mL human insulin (Beyotime, P3376) and 2 μM rosiglitazone (Selleck, S2556) in growth medium. After 2 days, the medium was replaced with medium containing the above concentration of insulin for another two days.

#### 3T3-L1 cell differentiation

3T3-L1 cells were cultured in growth medium (DMEM containing 10% NBCS and 1% penicillin‒streptomycin), and adipogenic induction was performed following the aforementioned protocol for primary preadipocytes. Lipid droplet formation was visualized with a microscope or by Oil Red O staining.

The cytotoxicity of palmitic acid (MCE, HY-N0830) toward primary differentiated adipocytes and of palmitic acid and/or ferrostatin-1 (MCE, HY-100579) toward 3T3-L1 adipocytes was assessed via the CCK-8 assay (Beyotime, C0039).

### FACS analysis

#### Flow staining

SVFs were incubated in PBS with the fixable viability dye eFluor® 780 (65-0865-14, eBioscience) for 30 min at 4 °C before antibody staining. Prior to surface staining, SVFs were incubated with an anti-CD16/CD32 antibody (BioLegend, 101320) to block Fc gamma receptors for 20 min at 4 °C. Thereafter, the cells were incubated with the appropriate primary antibodies diluted in FACS buffer (DPBS + 2% FCS) for 30 min at 4 °C. For CD206 staining, the cells were fixed with intracellular fixation and permeabilization buffer (Invitrogen, 88–8824) following the manufacturer’s instructions and subsequently incubated with antibodies for 30 min at 4 °C. For intracellular cytokine staining, the cells were first incubated in vitro with a cell activation cocktail (with brefeldin A) (BioLegend, 423303) in 10% FBS-RPMI for 4 h at 5% CO_2_ and 37 °C. Following cell surface staining, fixation, permeabilization, and intracellular staining were performed.

#### Detection and analysis

Data were acquired on a BD Canto II analyzer, BD LSRFortessa or CytoFlex LX (Beckman Coulter) and analyzed via FlowJo software (version 10, BD).

#### Antibodies and reagents

Anti-CD16/CD32 FC block (2.4G2), anti-CD45 (30-F11), anti-CD3 (145-2C11), anti-CD8a (53-6.7), anti-NK1.1 (PK136), anti-CD11c (N418), anti-CD11b (M1/70), anti-CD206 (C068C2), anti-CD69 (H1.2F3), anti-F4/80 (BM8), anti-TCRβ (H57-597), anti-TCRγ/δ (GL3), anti-TNFα (MP6-XT22), and cell activation cocktail (with brefeldin A) were purchased from Biolegend. Anti-IFN gamma (XMG1.2) was purchased from eBioscience.

### Glucose tolerance test and insulin tolerance test

#### IPGTT

For the IPGTT, the mice were fasted for 16 h before the administration of 2 grams per kilogram body weight of glucose (SIGMA, G7021) via intraperitoneal injection, and then, blood was collected from the tail vein at 0, 15, 30, 60, 90 and 120 min for blood glucose level measurements. Upon completion of the test, the mice were immediately provided free access to food and allowed at least 48 hours of recovery before subsequent experimental procedures.

#### IPITT

For the IPITT, the mice were fasted for 4 h before the administration of insulin (Beyotime, P3376). The insulin dose was administered at doses of 0.5 or 0.75 U per kg body weight in different experimental settings. Following insulin administration, blood samples were collected from the tail vein at 0, 15, 30, 45 and 60 min for blood glucose level measurements. Upon completion of the test, the mice were immediately provided free access to food and allowed at least 48 hours of recovery before subsequent experimental procedures.

### Serum lipid levels

Serum was collected by centrifugation at 2000 rpm at 4 °C for 15 min to measure the levels of total TC, TG, HDL, and LDL.

### Histological analyses

#### HE staining

Adipose tissues were collected and fixed in fatty tissue fixative solution for 24 hr, while other tissues were collected and fixed in 4% formalin. Following paraffin embedding and sectioning, the tissues were stained with hematoxylin and eosin. The size of the adipocytes was measured via ImageJ software. The hepatic histopathological changes were assessed via the MASLD scoring system and scoring of steatosis (0–3), ballooning (0–2), and lobular inflammation (0–3).

#### Oil red O staining

For Oil Red O staining, O.C.T.-embedded frozen liver sections were fixed in 4% paraformaldehyde, rinsed with 60% isopropanol and then stained with freshly prepared Oil Red O working solution. Oil Red O staining was quantitatively analyzed via ImageJ software.

### Immunohistochemical staining

Immunohistochemical analysis of B2M expression was performed on paraffin-embedded adipose tissue sections with an anti-B2M antibody (Abcam, ab218230, 1:500). In brief, adipose tissue sections were incubated at 4 °C overnight with primary antibodies against B2M, followed by incubation with horseradish peroxidase (HRP)-conjugated goat anti-mouse IgG (H + L) secondary antibodies (Servicebio, GB23301, 1:1000). The sections were subsequently visualized with DAB (Servicebio, G1212).

### Immunofluorescence staining

Costaining of F4/80 (Aifang, AFRM0005), CD206 (Aifang, AFRM0009), CD11c (Aifang, AFRM0081), 4-HNE (Bioss, bs-6313R) and Perilipin-1 (CST, 9349) was performed by Aifang Biotechnology Co., Ltd. (Hunan, China) via a five-color multiple immunofluorescence Kit (Aifang, AFIHC036).

Costaining of B2M (Abcam, ab218230), HFE (ABconal, A1310), TFR2 (ABconal, A9845), TFR1 (ABconal, A5865) and Perilipin-1 (CST, 9349) was performed by Aifang Biotechnology Co., Ltd. (Hunan, China) via a five-color multiple immunofluorescence Kit (Aifang, AFIHC036).

### ROS staining in adipose tissue

Dihydroethidium (DHE, Sigma, D7008) was used to detect intracellular ROS in the EpiWAT. The cryosections were incubated with 10 μM DHE at 37 °C for 30 min and then stained with DAPI. DHE staining was visualized via confocal microscopy. The average fluorescence intensity of the ROS was measured via ImageJ software.

### TUNEL staining of adipose tissue

For formalin-fixed paraffin-embedded EpiWAT samples, after blocking, the samples were incubated with Perilipin-1 antibody (1:100, CST, 9349, prepared in PBS containing 3% BSA) overnight at 4 °C. The bound antibodies were detected with Alexa Fluor 488-conjugated anti-ribbit IgG (1:200, Servicebio, GB25303) in PBS containing 3% BSA. The samples were incubated in the TUNEL reaction mixture from the TUNEL Cell Apoptosis Detection Kit (Servicebio, G1502) for 1 hr at 37 °C, after which the cell nuclei were counterstained with DAPI. The samples were photographed via a fluorescence microscope.

### Measurement of iron, ferrous ion, GSH and MDA contents in adipoc,ytes

The intracellular iron, ferrous ion, GSH and MDA concentrations were detected via a tissue iron content assay kit, a ferrous ion content assay kit (Solarbio, BC5415), a reduced glutathione (GSH) assay kit (Solarbio, BC1175) and a malondialdehyde (MDA) assay kit (Solarbio, BC0025), respectively, according to the manufacturers’ protocols.

#### Lipid peroxide, ROS, and Fe^2+^ measurements

The C11-BODIPY 581/591 probe (MCE, D1301), DCFH-DA probe (Meilunbio, MA0219) and FerroOrange probe (Elabscience, E-BC-F101) were used to investigate the production of lipid peroxides, ROS and ferrous ions (Fe^2+^) in 3T3-L1 adipocytes or primary differentiated adipocytes subjected to different treatments, respectively, according to the manufacturers’ protocols.

### RNA extraction and quantitative real-time PCR (qPCR)

Total RNA from target cells was isolated via the SteadyPure Quick RNA Extraction Kit (Accurate Biology, AG21023) according to the manufacturer’s instructions. To isolate RNA from WAT, TRIzol reagent (ABconal, RK30129) was used. Complementary DNA was synthesized via the *Evo M-MLV*RT Mix Kit (Accurate Biology, AG11728), and qPCR was performed via the Premix *Pro Taq* HS qPCR Kit (Accurate Biology, AG11701). The mRNA expression levels of the target genes were normalized to that of *18S rRNA*. The primers used are listed in Supplementary Table 2.

### Western blotting

#### Protein extraction and Western blotting

Protein was extracted by homogenizing tissue or cell samples in RIPA buffer (Beyotime, P0013B) supplemented with a protease inhibitor (MCE, HY-K0010). To analyze proteins in cell membranes, the membrane components were separated via a membrane and cytosol protein extraction kit (Beyotime, P0033) according to the manufacturer’s instructions. Protein concentrations were measured via a BCA protein assay kit (ABconal, RM02867). Proteins were separated via FuturePAGE™ 4‒20% protein pregel (ACE, ET15420LGel) and then transferred to PVDF membranes (0.2 μm, Millipore, ISEQ00010). After the membranes were blocked in 5% skim milk (Beyotime, P0216) for 1 h at room temperature, they were incubated overnight at 4 °C with primary antibodies and then for 40 min at room temperature with the corresponding secondary antibodies. A ChemiDoc MP Imaging System (Bio-Rad) was used for signal detection. Protein expression was quantified via ImageJ software and normalized to the levels of the corresponding internal controls.

#### Antibodies

Anti-CD71 antibody (1:2000 dilution; ABconal, A5865), anti-ferritin heavy chain antibody (1:3000 dilution; ABconal, A19544), anti-ferritin light chain antibody (1:3000 dilution; proteintech, 10727-1-AP), anti-HFE antibody (1:1000 dilution; ABconal, A1310), anti-HAMP antibody (1:1000 dilution; Invitrogen, PA5-102436), anti-HLA class 1 ABC antibody (1:1000 dilution; Huaan Biotechnology Co., Ltd., EM1801-10), anti-FPN1 antibody (1:1000 dilution; proteintech, 26601-1-AP), anti-TFR2 antibody (1:1000 dilution; ABconal, A9845), anti-beta 2 microglobulin antibody (1:1000 dilution; Abcam, ab218230), beta-actin monoclonal antibody (1:20,000 dilution; proteintech, 66009-1-lg), and anti-vinculin antibody (1:3000 dilution; proteintech, 66305-1.

### Coimmunoprecipitation (Co-IP)

Mature adipocytes were extracted as described above, and the membrane components of mature adipocytes were separated via a membrane and cytosol protein extraction kit (Beyotime, P0033) according to the manufacturer’s instructions. Co-IP was performed using an immunoprecipitation kit (Beyotime, P2179M). The samples were analyzed via Western blotting with anti-CD71 antibody (ABconal, A5865), anti-HFE antibody (Santa Cruz, sc-514405), anti-TFR2 antibody (ABconal, A9845), anti-beta 2 microglobulin antibody (Abcam, ab218230) and anti-Na, K-ATPase antibody (CST, 3010).

### Transmission electron microscopy (TEM)

Fresh epididymal fat pads were quickly harvested, transferred to precooled fixative for TEM, cut into small pieces of no more than 1 mm^3^, and fixed in fresh TEM fixative at room temperature for 2 h before being stored at 4 °C. The tissue pieces were sent to Servicebio (Wuhan, China) for subsequent sectioning and image processing. Electron microscopy images were acquired on an HT7800 transmission electron microscope (Hitachi).

### Serum cytokine concentrations

Serum cytokines (including insulin, adiponectin, leptin, glucagon, TNFα, INFγ, IL-6 and IL-1β) were measured via ELISA kits (Jingmei Biotechnology) according to the manufacturer’s instructions. The ELISA kit catalog numbers are as follows: JM-02862M1 (insulin), JM-02830M1 (adiponectin), JM-02902M1 (leptin), JM-02450M1 (glucagon), JM-02415M1 (TNF-α), JM-02465M1 (INFγ), JM-02446M2 (IL-6), and JM-0 2323M1 (IL-1β).

### In vitro coculture of adipocytes and immune cells

#### Primary adipocyte pretreatment

Primary preadipocytes from NCD-fed *B2m*^f/f^ or *B2m*^cKO^ mice were cultured and differentiated into adipocytes as described above. Adipocytes were exposed to PA (2.5 mM) and/or Fer-1 (20 μM) for 24 hr, followed by replacement with fresh medium for subsequent coculture experiments.

#### In vitro coculture experiment with primary adipocytes and ATMs

ATMs were isolated from the EpiWAT of NCD-fed *B2m*^f/f^ mice and sorted with magnetic beads (130-110-443, Miltenyi Biotec). Following the abovementioned drug administration, primary differentiated adipocytes from *B2m*^f/f^ or *B2m*^cKO mice^ were cultured with purified ATMs for 24 hours. The expression of CD80, CD86 and CD11c in CD45^+^F,4/80^+^ ATMs was detected by flow cytometry. The antibodies used for flow cytometry were as follows. Anti-CD45 (30-F11), anti-F4/80 (BM8) and anti-CD11c (N4,18) antibodies were purchased from Biolegend. Anti-CD80 (16-10A1) and anti-CD86 (GL1) antibodies were purchased from eBioscience.

#### In vitro coculture experiment with 3T3-L1 adipocytes and RAW264.7 macrophages

The coculture experiment involving 3T3-L1 adipocytes and RAW264.7 macrophages was performed following the protocol for the coculture of primary adipocytes and ATMs. Prior to coculture, 3T3-L1 adipocytes were subjected to adipogenic induction and treated with PA (2.5 mM) and/or Fer-1 (20 μM) for 24 hr. Subsequently, 3T3-L1 adipocytes and RAW264.7 macrophages were cocultured in fresh medium for an additional 24-h period. RAW264.7 macrophages were subsequently collected, and the expression levels of CD80, CD86, and CD11c in RAW264.7 macrophages were quantified via flow cytometry analysis.

#### In vitro coculture experiment with primary adipocytes and CD8^+^ T cells

CD8^+^ T cells were isolated from the EpiWAT of HFD-fed *B2m*^f/f^ mice via magnetic beads (130-104-075, Miltenyi Biotec). Prior to coculture, primary preadipocytes were subjected to adipogenic induction and treated with PA (2.5 mM) and/or Fer-1 (20 μM) for 24 hr. Subsequently, primary adipocytes and CD8^+^ T cells were cocultured in fresh medium and divided into two groups: direct contact coculture and Transwell chamber-separated culture (Labselect, 14212). For the transwell chamber-separated culture, adipocytes were seeded in the lower chamber, and CD8^+^ T cells were seeded in the upper chamber. After an 8-h coculture period, CD8^+^ T cells were collected, and the expression of CD69 (H1.2F3) on CD8^+^ T cells was evaluated via flow cytometry analysis. Before coculture, CD8^+^ T cells were labeled with CFSE (Invitrogen, C34554) to monitor proliferation. Following a 72-h coculture of CFSE-labeled CD8^+^ T cells with primary adipocytes, the CD8^+^ T cells were harvested, and CFSE retention was assessed via flow cytometry.

### RNA-Seq

The RNA from the samples was isolated and purified via TRIzol (ABconal) following the manufacturer’s protocol. The quantity and purity of the total RNA were assessed with a NanoDrop ND-1000 (NanoDrop, Wilmington, DE, USA), while the integrity of the RNA was evaluated with a Bioanalyzer 2100 (Agilent, CA, USA). The samples with concentrations >50 ng/μL, RIN values > 7.0 and total RNA concentrations >1 μg met the requirements for downstream experiments. Oligo(dT) magnetic beads (Dynabeads Oligo(dT), cat. 25–61005; Thermo Fisher Scientific, Inc., USA) were utilized to specifically capture mRNA containing PolyA through two rounds of purification. The captured mRNA underwent fragmentation via the NEBNext^R^ RNA Fragmentation Module (cat. E6150S, USA) under high-temperature conditions at 94 °C for 5‒7 min. cDNA synthesis from fragmented RNA was performed via Invitrogen SuperScript^TM^ II Reverse Transcriptase (cat. 1896649, CA, USA). *E. coli* DNA polymerase I (NEB, cat. m0209, USA) and RNase H (NEB, cat. m0297, USA) were used for double-stranded synthesis, followed by conversion into DNA double strands from complex double strands of DNA and RNA. Additionally, dUTP solution (Thermo Fisher, cat. R0133, CA, USA) was added to the double-stranded DNA; a flat end supplemented each ewas nd of the double-stranded DNA with an A base so that it could be connected to a terminal with a T base. The fragment size was screened, and the fragments were purified with magnetic beads. The two DNA strands were digested via the UDG enzyme (NEB, cat. no. M0280, Massachusetts, USA). PCR was subsequently performed with initial denaturation at 95 °C for 3 min, followed by 8 cycles of denaturation at 98 °C for 15 seconds, annealing at 60 °C for 15 seconds, and extension at 72 °C for 30 seconds. A final extension was carried out at 72 °C for 5 min. This process resulted in the formation of a library with a fragment size of 300 bp ± 50 bp (strand-specific library). The samples were ultimately subjected to double-ended sequencing via the Illumina^NovaSeqTM^ 6000 platform in PE150 mode, following standard protocols.

The raw data were subjected to QC analysis via FastQC v.0.11.9. The reads were then mapped to the mouse genome (mm10) via HISAT2 v.2.2.1. Transcriptome assembly and quantification were conducted with StringTie v.2.1.6, employing default parameters. Differential gene expression analysis was carried out via DESeq2 v.1.22.2, with a threshold of *q* < 0.05 for identifying differentially expressed genes (DEGs). RNA-seq was performed by Biotree (Shanghai, China). These identified differentially expressed genes were further utilized for downstream analyses, including gene set enrichment analysis (GSEA), differential gene analysis, and enrichment analysis, with results visualized through various approaches.

### Reanalysis of public RNA-seq data

The human SAT and VAT RNA-seq datasets reanalyzed in this study were derived from the GEO database under accession numbers GSE152991 and GSE286454, respectively.

### Statistics

All the data are presented as the means ± SDs, and the sample sizes are reported in the figure legends. Two-tailed Student’s *t* test or the Mann‒Whitney *U* test was used to compare the differences between two groups. One-way, two-way, or multiway ANOVA followed by Tukey’s multiple comparison test was performed to analyze multiple groups. Statistical analysis was performed via GraphPad Prism v.8.0.3. Statistical significance is represented as follows: *p < 0.05, **p < 0.01, ***p < 0.001.

## Supplementary information

Supplement Materials

Original Images of Representative Western Blot

## Data Availability

All the data needed to evaluate the conclusions in the article are presented in the article and/or the Supplementary Materials. All original source data linked to the figures in the manuscript: 10.6084/m9.figshare.30373963. The RNA-seq data generated and analyzed for this study have been archived in the NCBI SAR database PRJNA1204472.
