# Supplementary material for: Adipocytes orchestrate obesity-related chronic inflammation through β2-microglobulin
Source: Signal Transduct Target Ther. 2025 Dec 3;10:394. doi: 10.1038/s41392-025-02486-3 (PMC12672573; doi:10.1038/s41392-025-02486-3)
Supplement: Supplementary file 1 — Supplement Materials [file 41392_2025_2486_MOESM1_ESM.docx]

Supplement Materials for

**Adipocytes orchestrate obesity-related chronic inflammation through β2-microglobulin**

Jie Li^1,#^, Yuhao Li^1, #^, Xiaoyang Zhou^2, #^, Shushu Yang^1^, Dong Liu^1^, Hao Wen^1^, Xiaoling Chen^1^, Chengjie Duan^1^, Meiling Yu^1^, Mengjun Zhang^3^, Bo Tang^4^, Yong Wang^5,*^, Li Wang^1,7,*^ and Yuzhang Wu^1,*^

Correspondence to:

Li Wang, Department of Immunology, College of Basic Medicine, Army Medical University (Third Military Medical University), Chongqing 400038, China. E-mail: [liwang118@tmmu.edu.cn](mailto:liwang118@tmmu.edu.cn).

Yuzhang Wu, Department of Immunology, College of Basic Medicine, Army Medical University (Third Military Medical University), Chongqing 400038, China. E-mail: [wuyuzhang@tmmu.edu.cn](mailto:wuyuzhang@tmmu.edu.cn).

Yong Wang, Department of Laboratory Animal Science, College of basic medical sciences, Army Medical University, Chongqing 400038, China. E-mail: [yongwanglas@tmmu.edu.cn](mailto:yongwanglas@tmmu.edu.cn).

^#^These authors contributed equally: Jie Li, Yuhao Li, Xiaoyang Zhou

^7^Lead contact

**This file includes:**

Figures. S1 to S7

Tables S1 to S2

**Other Supplementary Materials for this manuscript include the following:**

Original Images of Representative Western blot

Original data for linked to the figures

**
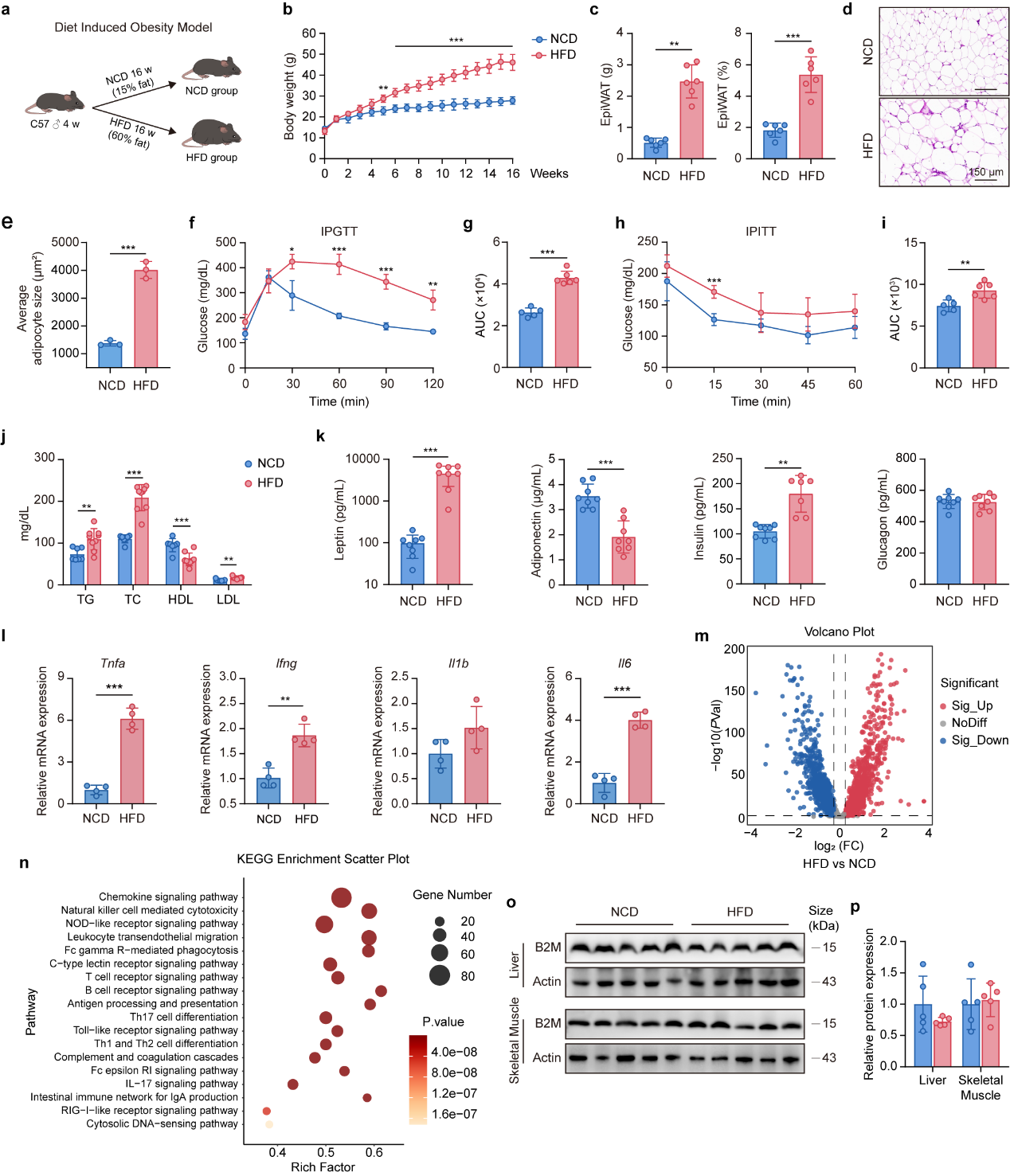
Figure. S1. Establishment of a diet-induced obesity model. a** Schematic representation of the diet-induced obesity model. **b-c** Body weights (**b**) and the weight and proportion (**c**) of EpiWAT of NCD- and HFD-fed mice (*n* = 6). **d-e** H&E staining of EpiWAT and quantitation of average adipocyte size (*n* = 3). Scale bar, 150 μm. **f-i** IPGTT (2 g glucose per kg body weight), IPITT (0.5 U insulin per kg body weight) and their respective AUC (*n* = 5-6). **j** TG, TC, HDL and LDL (*n* = 7-9). **k** Serum leptin, adiponectin, insulin and glucagon levels in NCD- and HFD-fed mice after 4 hours of fasting (*n* = 7-8). **l** Relative mRNA levels of *Tnfa, Ifng*, *Il1b,* and *Il6* in EpiWAT (*n* = 4). **m** Volcano plot of the DEGs in mature adipocytes from HFD-fed vs. NCD-fed mice. **n** Bubble diagram showing the enriched immune-related KEGG pathways of DEGs in mature adipocytes from NCD- and HFD-fed mice. **o-p** Protein expressions and relative quantification in liver and skeletal muscle (*n* = 4). The data are representative as the mean ± standard deviation (SD), with “n” representing the number of biological replicates per experimental group. Significant in **c**, **e**, **g**, **i**-**l**, and **p** was calculated using a two-tailed Student’s *t* test. Significant in **b**, **f,** and **h** was calculated using two-way ANOVA followed by Tukey’s HSD post hoc test for multiple comparisons. **p* < 0.05, ***p* < 0.01, ****p* < 0.001. AUC, area under the curve, DEGs, differentially expressed gene HFD, high-fat diet, s, EpiWAT, epididymal adipose tissue, HDL, high density lipoprotein, IPGTT, Intraperitoneal glucose tolerance test, IPITT, intraperitoneal insulin tolerance test, LDL, low density lipoprotein, NCD, normal chow diet, TC, cholesterol, TG, triglyceride.

**
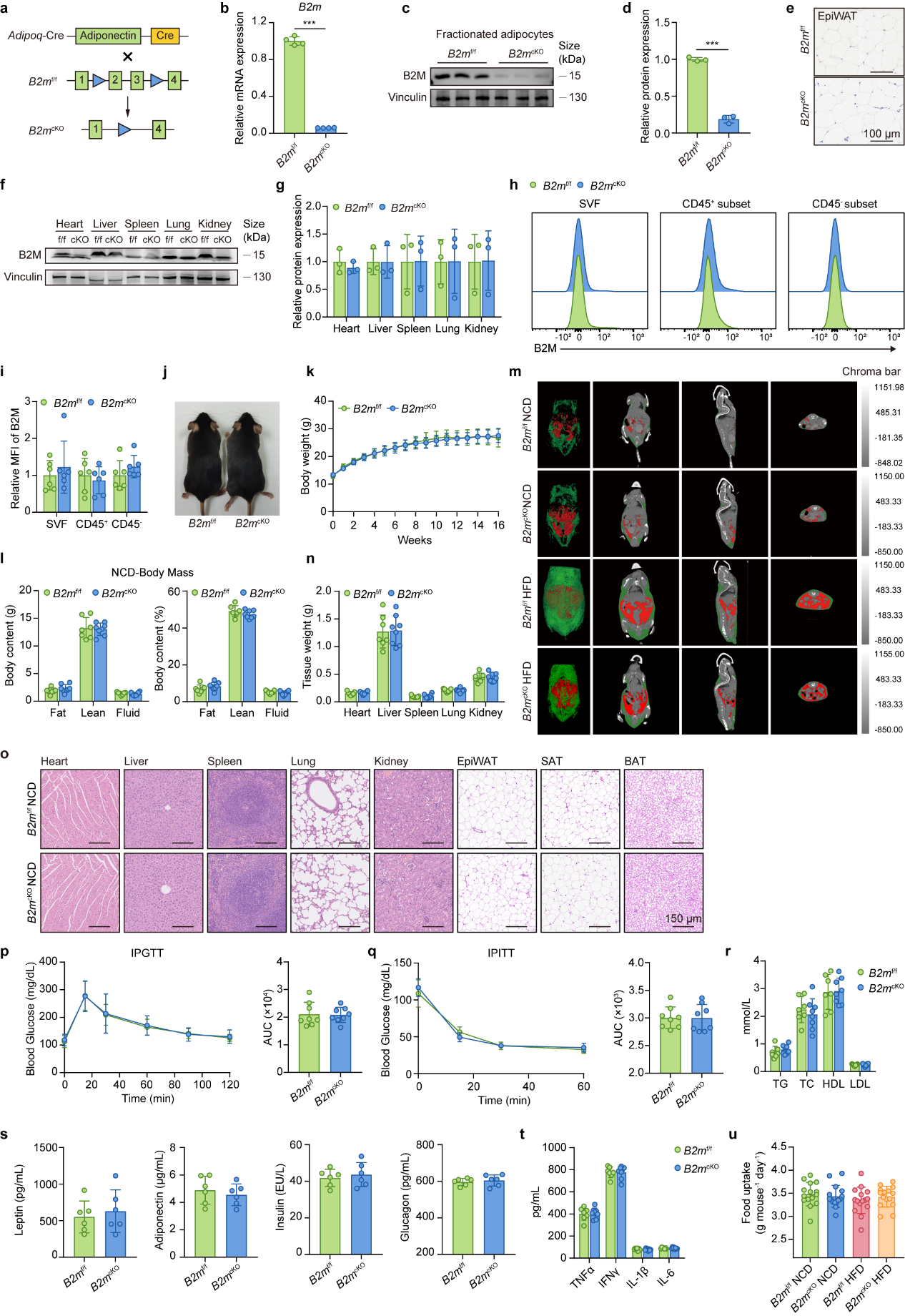
**

**Figure. S2. Adipocyte-specific B2M knockout does not change the metabolic phenotype of mice fed on NCD. a** Schematic illustration of the targeting strategy to generate adipocyte-specific B2M knockout (*B2m*^cKO^) mice. **b-d** Relative *B2m* mRNA levels (**b**), protein expressions (**c**) and relative quantification (**d**) in the mature adipocyte fraction isolated from EpiWAT (*n* = 3-4). **e** Representative images of B2M immunostaining in EpiWAT. Scale bar, 100 μm. **f-g** Protein expression and relative quantitation of B2M in the indicated tissues (*n* = 3)*.* **h-i** Representative flow cytometry diagram and relative MFI analysis of B2M expression in the SVF, CD45^+^ and CD45^-^ cells from EpiWAT (*n* = 6)*.* **j** Representative photograph of *B2m*^f/f^ and *B2m*^cKO^ mice after 16 weeks on NCD. **k** Body weights (*n* = 7-8)*.* **l** Body content (fat, lean, water) as analyzed by nuclear magnetic resonance spectrometer (*n* = 7-8)*.* **m** Representative images of micro-computed tomography scanning (*n* = 3)*.* **n** The weights of Heart, Liver, Spleen, Lung and Kidney (*n* = 7-8)*.* **o** Representative images of histological H&E staining of indicated sections (*n* = 3). Scale bars, 150 μm. **p-q** IPGTT (2 g glucose per kg body weight), IPITT (0.75 U insulin per kg body weight) and their respective AUC (*n* = 8)*.* **r** Serum levels of TG, TC, HDL and LDL (*n* = 7-8)*.* **s** Serum levels of adiponectin, leptin, insulin and glucagon in NCD-fed *B2m*^f/f^ and *B2m*^cKO^ mice after 4 hours of fasting (*n* = 6)*.* **t** Serum levels of TNFα, IFNγ, IL-1β and IL-6 (*n* = 7-8)*.* **u** Daily food intake per mouse. The data are representative as the mean ± standard deviation (SD), with “n” representing the number of biological replicates per experimental group. Significant in **b**, **d**, **g**, **i**, **l**, **n**, AUC in **p**, AUC in **q**, and **r-t** was calculated using a two-tailed Student’s *t* test. Significant in **u** was calculated using two-way ANOVA followed by Tukey’s HSD post hoc test for multiple comparisons. Significant in **k**, **p,** and **q** was calculated using two-way ANOVA followed by Tukey’s HSD post hoc test for multiple comparisons. **p* < 0.05, ***p* < 0.01, ****p* < 0.001. AUC, area under the curve, BAT, brown adipose tissue, EpiWAT, epididymal adipose tissue, HDL, high density lipoprotein, HFD, high-fat diet, IPGTT, intraperitoneal glucose tolerance test, IPITT, intraperitoneal insulin tolerance test, LDL, low density lipoprotein, NCD, normal chow diet, SAT, subcutaneous adipose tissue, SVF, stromal vascular fraction, TC, cholesterol, TG, triglyceride.

**
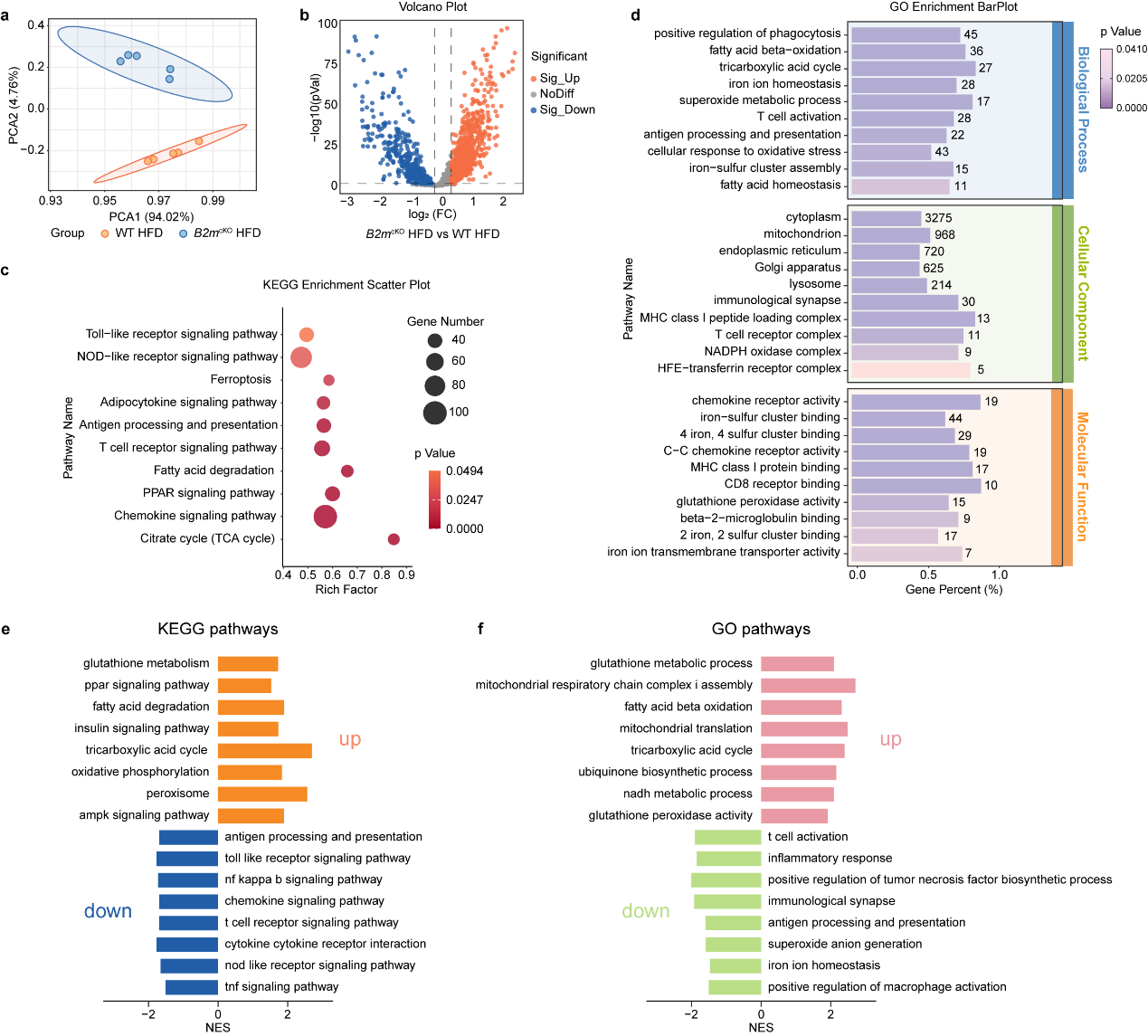
**

**Figure. S3. A comparative transcriptome analysis of mature adipocytes from EpiWAT of control and *B2m*^cKO^ mice fed HFD.** A transcriptome-wide analysis was conducted on mature adipocytes isolated from EpiWAT of male wild-type and *B2m*^cKO^ mice after being fed a 16-week HFD starting at the age of 4 weeks. **a** PCA. **b** Volcano plot of the DEGs in HFD-fed WT mice vs. HFD-fed *B2m*^cKO^ mice. **c** KEGG bubble diagram of selected enrichment pathways of DEGs. **d** GO bar chart of selected enrichment pathways of DEGs for biological process, molecular function, and cell component. **e-f** GSEA bar chart showing the up-regulated and down-regulated pathways identified through KEGG (**e**) and GO (**f**) analyses. DEGs, differentially expressed genes, EpiWAT, epididymal adipose tissue, HFD, high fat diet, PCA, principal component analysis.


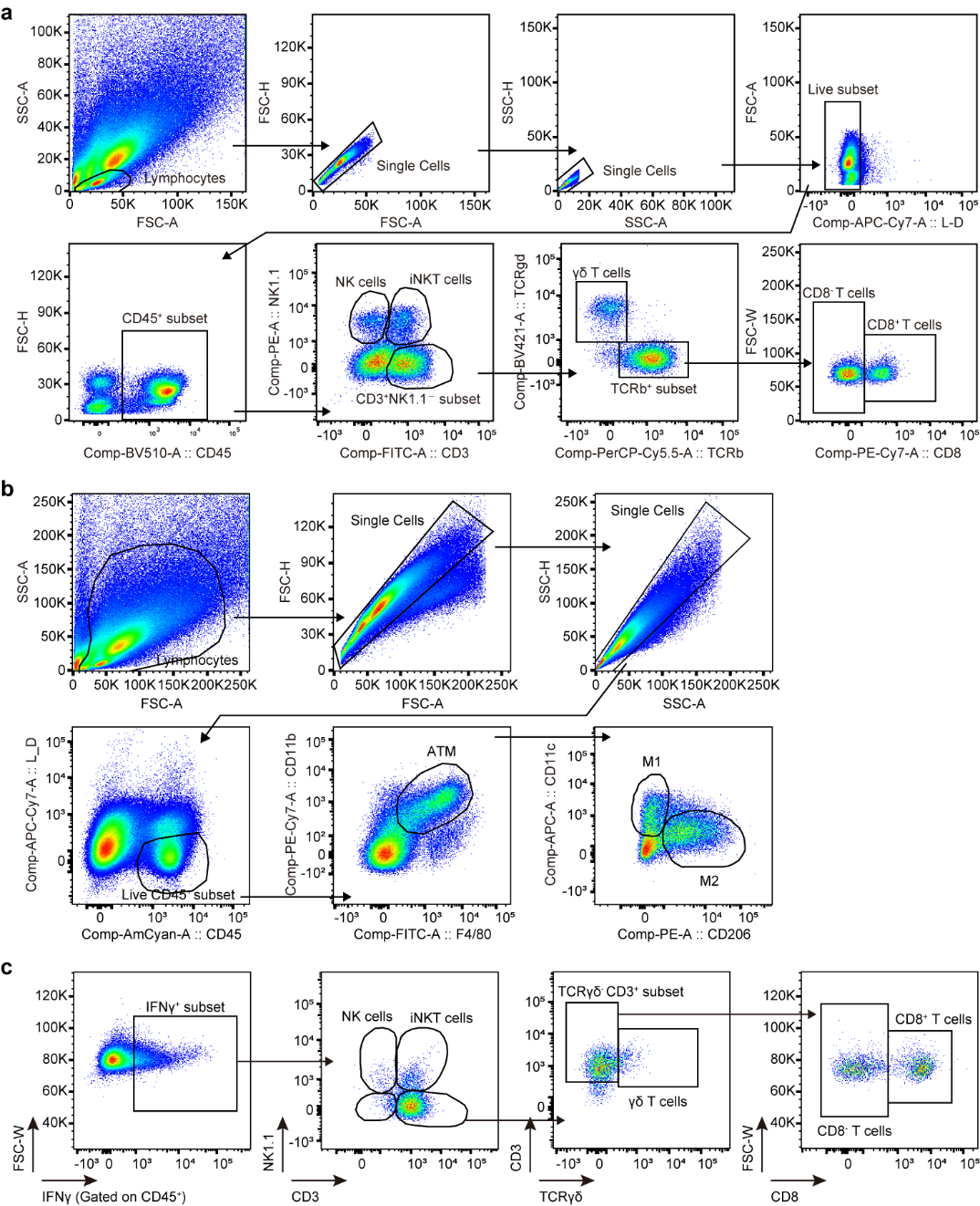


**Figure. S4. Gating Strategies for flow cytometry analysis of immune cells in adipose tissues. a** Representative flow cytometry plots for the analysis of CD8^+^ T cells, CD8^-^ T cells, γδ T cells, NK cells, and iNKT cells within the EpiWAT of mice. **b** Representative flow cytometry plots for the analysis of CD11b^+^F4/80^+^ ATMs, CD11b^+^F4/80^+^CD11c^+^CD206^-^ M1 and CD11b^+^F4/80^+^CD11c^-^CD206^+^ M2 within the EpiWAT of mice. **c** Representative flow cytometry plots for the analysis of CD8^+^ T cells, CD8^-^ T cells, γδ T cells, iNKT cells and others in IFNγ^+^ subset within the EpiWAT of mice. The IFNγ^+^ subset is gating on live CD45^+^ cells. ATMs, adipose tissue macrophages, EpiWAT, epididymal adipose tissue, iNKT, invariant natural killer T, NK, Natural killer.

**
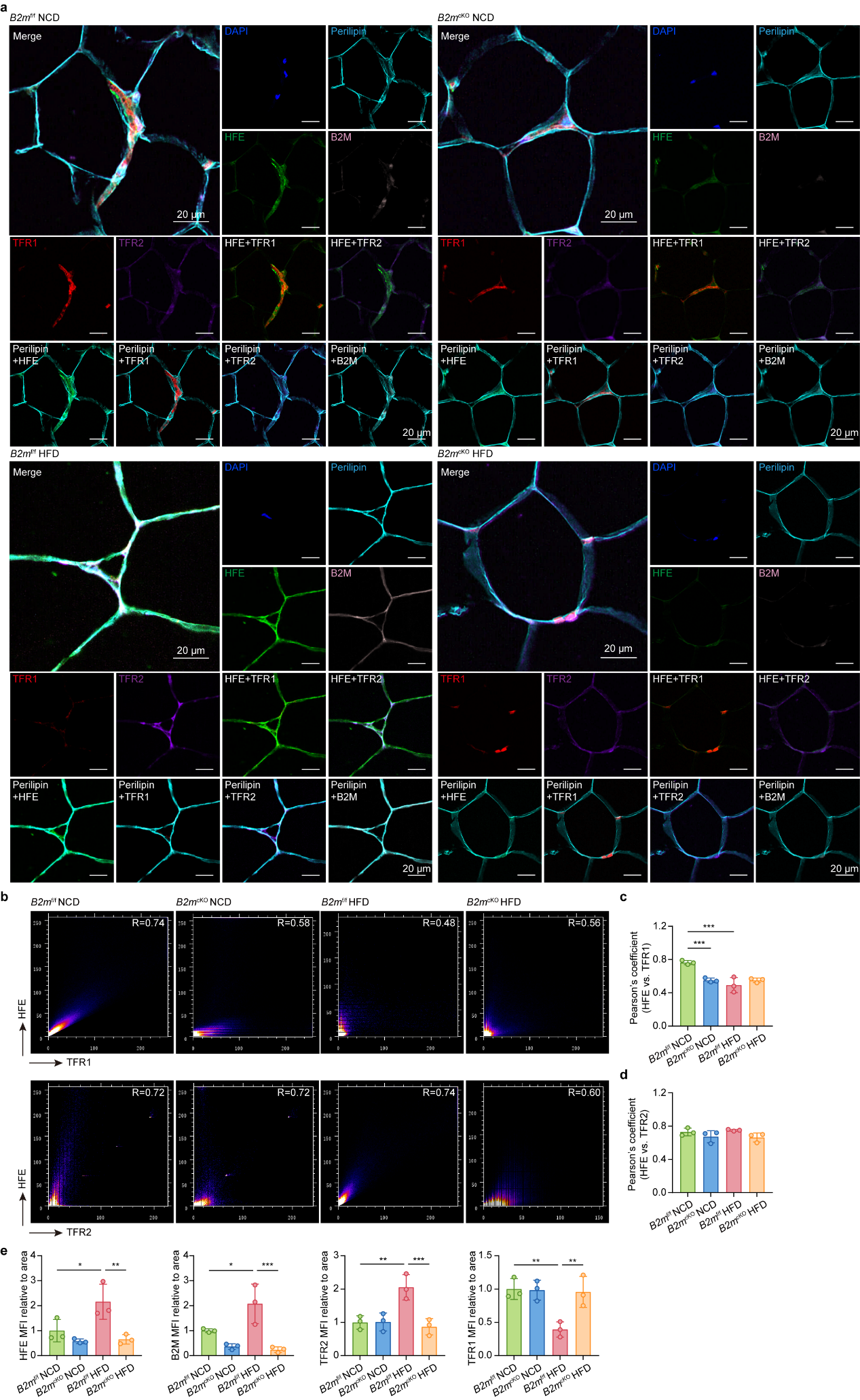
Figure. S5. HFD affects the colocalization between HFE with TFR1 and TFR2 on the surface of adipocytes. a** Representative immunofluorescence images of Perilipin (cyan), B2M (pink), HFE (green), TFR1 (red), TFR2 (purple), and DAPI (blue) in the sections of EpiWAT from *B2m*^f/f^ and *B2m*^cKO^ mice fed HFD or NCD for 16 weeks Scale bar, 20 μm. **b** Representative colocalization scatter plots of HFE with TFR1 (upper panel), and HFE with TFR2 (lower panel), with the Pearson correlation coefficient in the upper right corner. **c-d** Quantitation of the colocalization relationship between HFE and TFR1 (**c**), as well as between HFE and TFR2 (**d**), was measured by applying Pearson’s colocalization coefficient using Colocalization Finder plugin in Fiji (n=3). **e** Quantitation of the fluorescence intensity normalized to the field of view area (n=3). The data are representative as the mean ± standard deviation (SD), with “n” representing the number of biological replicates per experimental group. Significant in **c-e** was calculated using two-way ANOVA followed by Tukey’s HSD post hoc test for multiple comparisons. **p* < 0.05, ***p* < 0.01, ****p* < 0.001. EpiWAT, epididymal adipose tissue, HFD, high fat diet, NCD, normal chow diet.

**
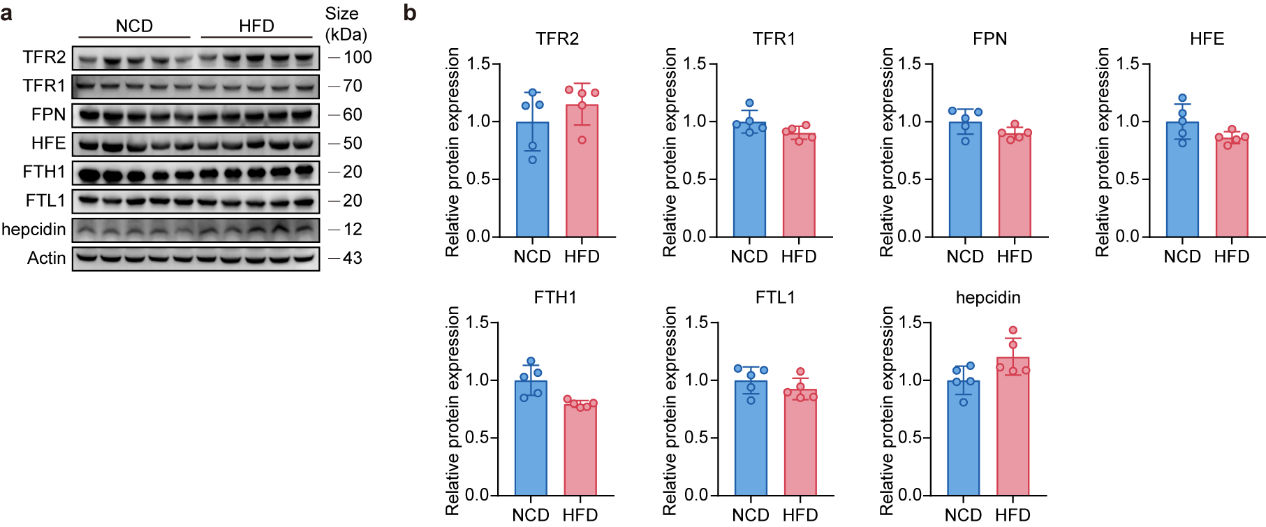
Figure. S6. No significant alterations of protein associated with iron transport and storage in the livers of 16-week HFD-fed mice. a-b** Protein expressions (**a**) and relative quantitation (**b**) of TFR2, TFR1, FPN, HFE, FTH1, FTL1 and Hepcidin in liver of NCD- or HFD-fed *B2m*^f/f^ and *B2m*^cKO^ mice (*n* = 5). The data are representative as the mean ± standard deviation (SD), with “n” representing the number of biological replicates per experimental group. Significant in **b** was calculated using a two-tailed Student’s *t* test. **p* < 0.05. NCD, normal chow diet, HFD, high fat diet.


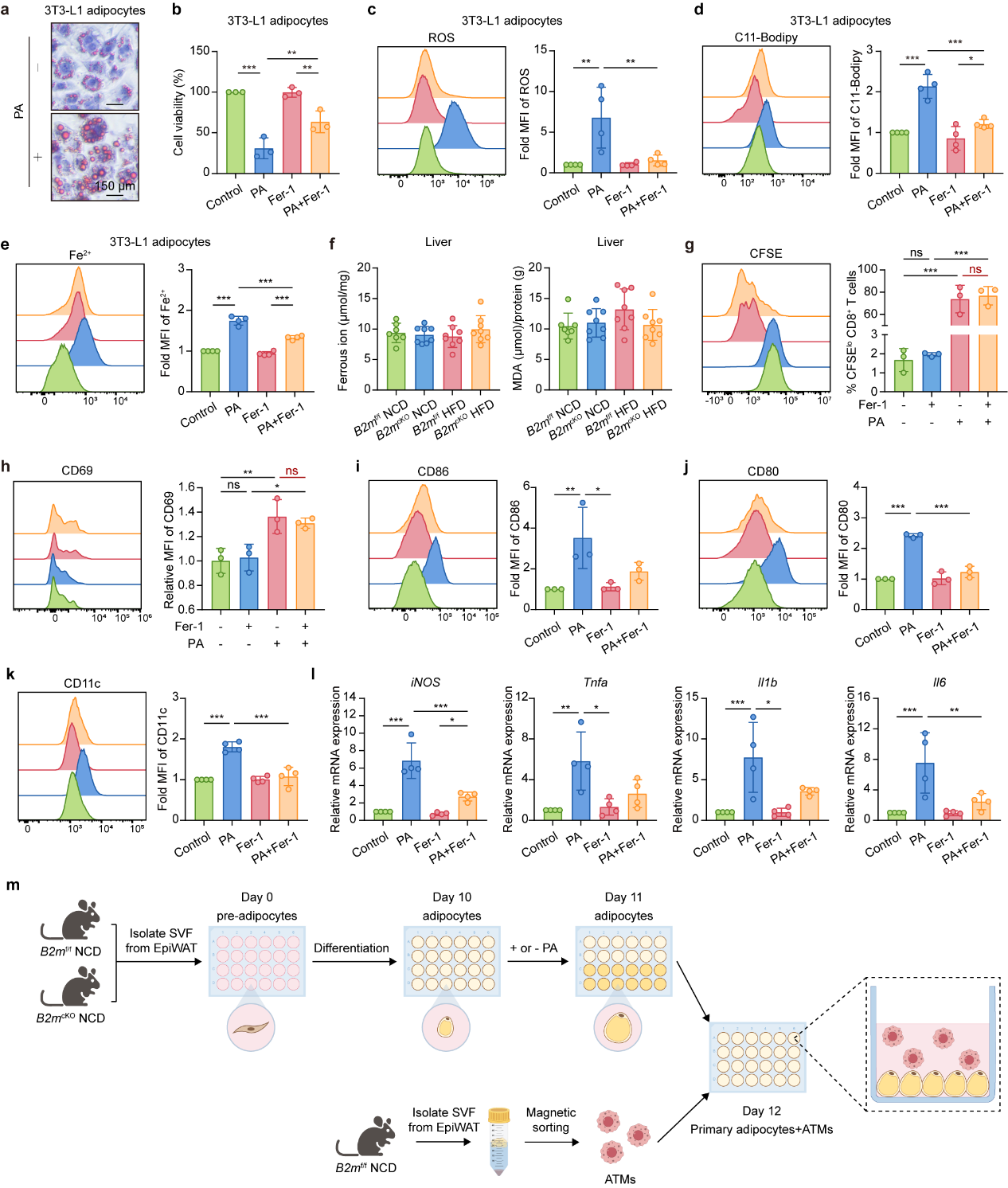

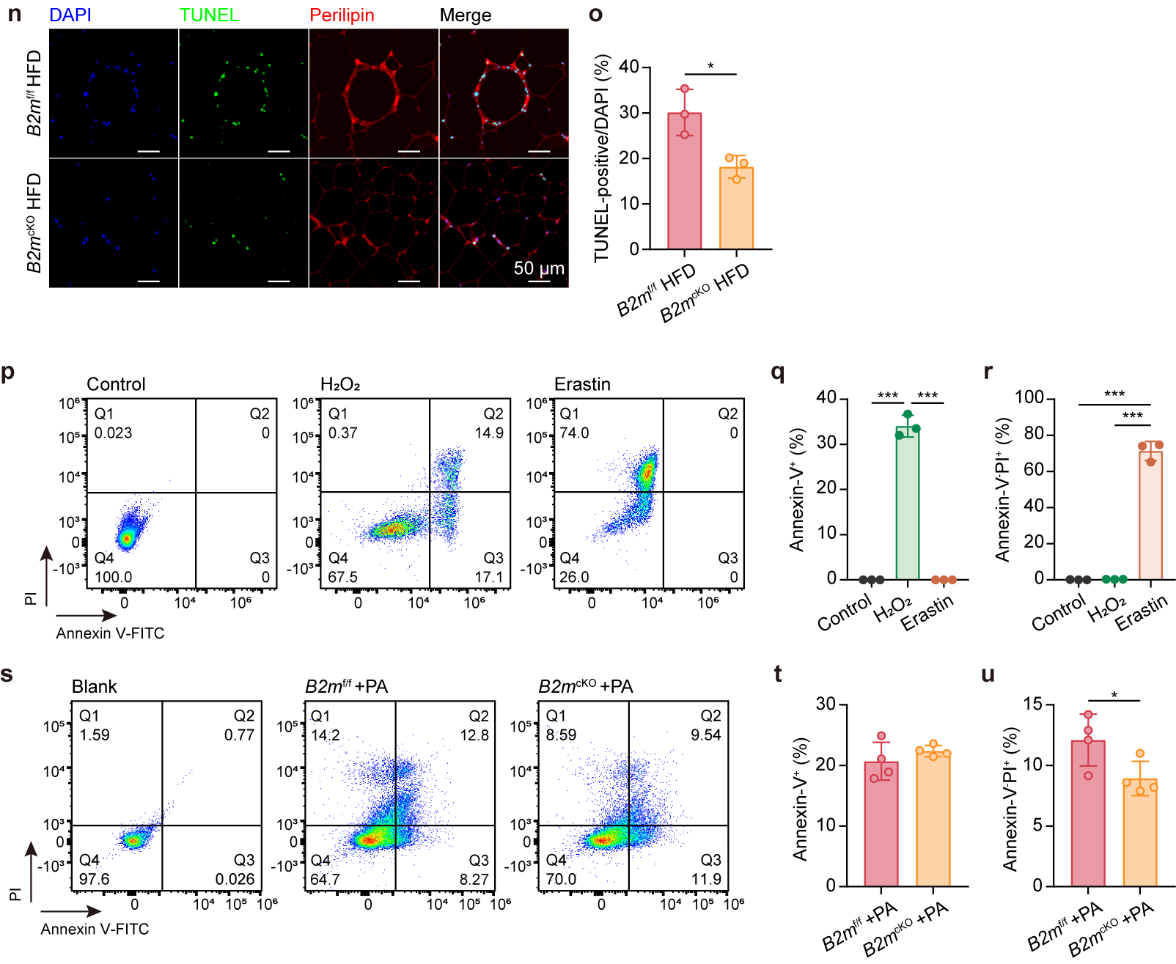


**Figure. S7. PA treatment induces ferroptosis in adipocytes and in turn promotes M1 polarization of macrophages in co-culture. a** 3T3-L1 cells were induced to differentiate into adipocytes and then treated with PA (2.5 mM) for 24 hours. Representative images of oil red O staining, Scale bar, 150 μm. **b-e** 3T3-L1 cells were induced to differentiate into adipocytes and then treated with PA (2.5 mM) and/or Fer-1 (20 μM) for 24 hours. Cell viability (**b**). The levels of ROS (**c**), lipid peroxides (**d**), and Fe^2+^ (**e**) (*n* = 3-4). **f** Ferrous ion content and MDA in liver of NCD- or HFD-fed *B2m*^f/f^ and *B2m*^cKO^ mice (*n* = 7-8). **g-h** CD8^+^ T cells were isolated from EpiWAT of obese *B2m*^f/f^ mice using a magnetic bead sorting kit and labeled with CFSE before co-culture. Adipocytes were induced from primary adipocyte precursors in SVF and treated with PA (2.5 mM) and/or Fer-1 (20 μM) for 24 hours before co-culture. Flow cytometric analysis of proliferation (**g**) and CD69 (**h**) expression in CD8^+^ T cells following co-culture with primary adipocytes. (*n* = 3). **i-k** Flow cytometry analysis of surface expression levels of CD86 (**g**), CD80 (**h**) and CD11c (**i**) in RAW264.7 cells co-cultured with 3T3-L1 adipocytes pre-treated with PA and/or Fer-1 for 24 hr (*n* = 3-4). **l** Relative mRNA levels of *iNOS*, *Tnfa*, *Il1b* and *Il6* of RAW264.7 cells co-cultured with 3T3-L1 adipocytes pre-treated with PA and/or Fer-1 for 24 h (*n* = 4). **m** Schematic representation of the in vitro co-culture experiment involving primary adipocytes with ATMs. ATMs were isolated from EpiWAT of lean *B2m*^f/f^ mice using a magnetic bead sorting kit. Primary adipocytes were differentiated from adipocyte precursors in SVF from EpiWAT of lean *B2m*^f/f^ or *B2m*^cKO^ mice and treated with or without PA for 24 hours before co-culture. **n-o** Representative images (**n**) and quantification (**o**) of TUNEL staining in EpiWAT of HFD-fed *B2m*^f/f^ and *B2m*^cKO^ mice, perilipin (red), TUNEL (green), DAPI (blue) (*n* = 3). Scale bar, 50 μm. **p-r** 3T3-L1 adipocytes were treated with the apoptosis inducer H₂O₂ (2 μM) and the ferroptosis inducer Erastin (2 μM) for 24 hours, respectively. Representative flow cytometry plots (**p**) and quantitation of Annexin-V^+^ (**q**) and Annexin-V^-^PI^+^ (**r**) ratio detected by flow cytometry (*n* = 3). **s-u** Adipocytes were induced from primary adipocyte precursors in SVF of NCD-fed *B2m*^f/f^ and *B2m*^cKO^ mice and treated with PA (1 mM) for 24 hours. Representative flow cytometry plots (**p**) and quantification of Annexin V⁺ (**t**) and Annexin-V^-^PI⁺ (**u**) rate (*n* = 4).The data are representative as the mean ± standard deviation (SD), with “n” representing the number of biological replicates per experimental group. Significant in **b-l** was calculated using two-way ANOVA followed by Tukey’s HSD post hoc test for multiple comparisons. Significant in **o**, **q**, **r** , **t** and **u** was calculated using a two-tailed Student’s *t* test.**p* < 0.05, ***p* < 0.01, ****p* < 0.001. ATMs, adipose tissue macrophages, EpiWAT, epididymal adipose tissue, Fer-1, ferrostatin-1, HFD, high fat diet, NCD, normal chow diet, MDA, malondialdehyde, PA, palmitic acid, ROS, reactive oxygen species, SVF, stromal vascular fraction.

|  | Lean (n = 3)  Mean ± SD | Obese (n = 3)  Mean ± SD |
| --- | --- | --- |
| Age (years) | 57.3 ± 11.32 | 49.0 ± 10.80 |
| BMI (kg/m^2^) | 20.7 ± 1.16 | 27.5 ± 1.56 |
| FPG (mg/dL) | 4.81 ± 0.69 | 8.62 ± 2.89 |
| *TG (mg/dL) | 0.98 ± 0.10 | 1.75 ± 0.26 |
| TC (mg/dL) | 4.26 ± 0.73 | 4.55 ± 0.30 |
| HDL (mg/dL) | 0.86 ± 0.09 | 0.82 ± 0.35 |
| LDL (mg/dL) | 2.58 ± 0.34 | 2.79 ± 0.21 |
| SBP (mmHg) | 124.67 ± 16.78 | 124.00 ± 9.93 |
| DBP (mmHg) | 76.67 ± 11.14 | 79.00 ± 7.87 |

**Table S1.** Phonotypic parameters of the human samples. Asterisks indicate significant differences (p-value <0.05 by two-tailed Student’s *t* test) between lean patients and patients with obesity. Body mass index (BMI), FPG, fasting plasma glucose, TG, triglyceride, TC, cholesterol, HDL, high density lipoprotein, LDL, low density lipoprotein, systolic and diastolic blood pressure (SBP and DBP). The data are representative as the mean ± standard deviation (SD), with “n” representing the number of subject population.

| Gene | Forward (5'-3') | Reverse (5'-3') |
| --- | --- | --- |
| *18S* | GTAACCCGTTGAACCCCATT | CCATCCAATCGGTAGTAGCG |
| *Adipoq* | GACGTTACTACAACTGAAGAGC | CATTCTTTTCCTGATACTGGTC |
| *B2m* | TTCTGGTGCTTGTCTCACTGA | CAGTATGTTCGGCTTCCCATTC |
| *Fth* | GGTGACCACGTGACCAACTT | CTCTCATCACCGTGTCCCAG |
| *Ftl* | ATGGGCAACCATCTGACCAA | TTGAGAGTGAGGCGCTCAAA |
| *H2-D1* | GTAAAGCGTGAAGACAGCTGC | CTGAACCCAAGCTCACAGG |
| *H2-K1* | CGGCGCTGATCACCAAACA | AGCGTCGCGTTCCCGTT |
| *Hamp* | ACAGCAGAACAGAAGGCATGATGG | TCAGGCTGGCAAGGAGGAGAAG |
| *Hfe* | CTGGCTTGAGGTTTGCTCC | CACCGCGTTCACATTCTCTAA |
| *Ifng* | AGACAATCAGGCCATCAGCAA | CTCATTGAATGCTTGGCGCT |
| *Il1b* | GAAATGCCACCTTTTGACAGTG | TGGATGCTCTCATCAGGACAG |
| *Il6* | TAGTCCTTCCTACCCCAATTTCC | TTGGTCCTTAGCCACTCCTTC |
| *iNOS* | ACTCAGCCAAGCCCTCA | CTCTGCCTATCCGTCTCGT |
| *Lep* | TGAGTTTGTCCAAGATGGACC | GCCATCCAGGCTCTCTGG |
| *Tfr1* | CTCAGTTTCCGCCATCTCAGT | GCAGCTCTTGAGATTGTTTGCA |
| *Tfr2* | CTAGACTTCGGCCGCTATGG | CCCTTGCAGAGTACACCCAC |
| *Tnfa* | GAGAAAGTCAACCTCCTCTCTG | GAAGACTCCTCCCAGGTATATG |

**Table S2.** List of primers used in the qPCR assay.
