# Supplementary figures and images for: Adipocytes orchestrate obesity-related chronic inflammation through β2-microglobulin
Source: Signal Transduct Target Ther. 2025 Dec 3;10:394. doi: 10.1038/s41392-025-02486-3 (PMC12672573; doi:10.1038/s41392-025-02486-3)

Original Blots


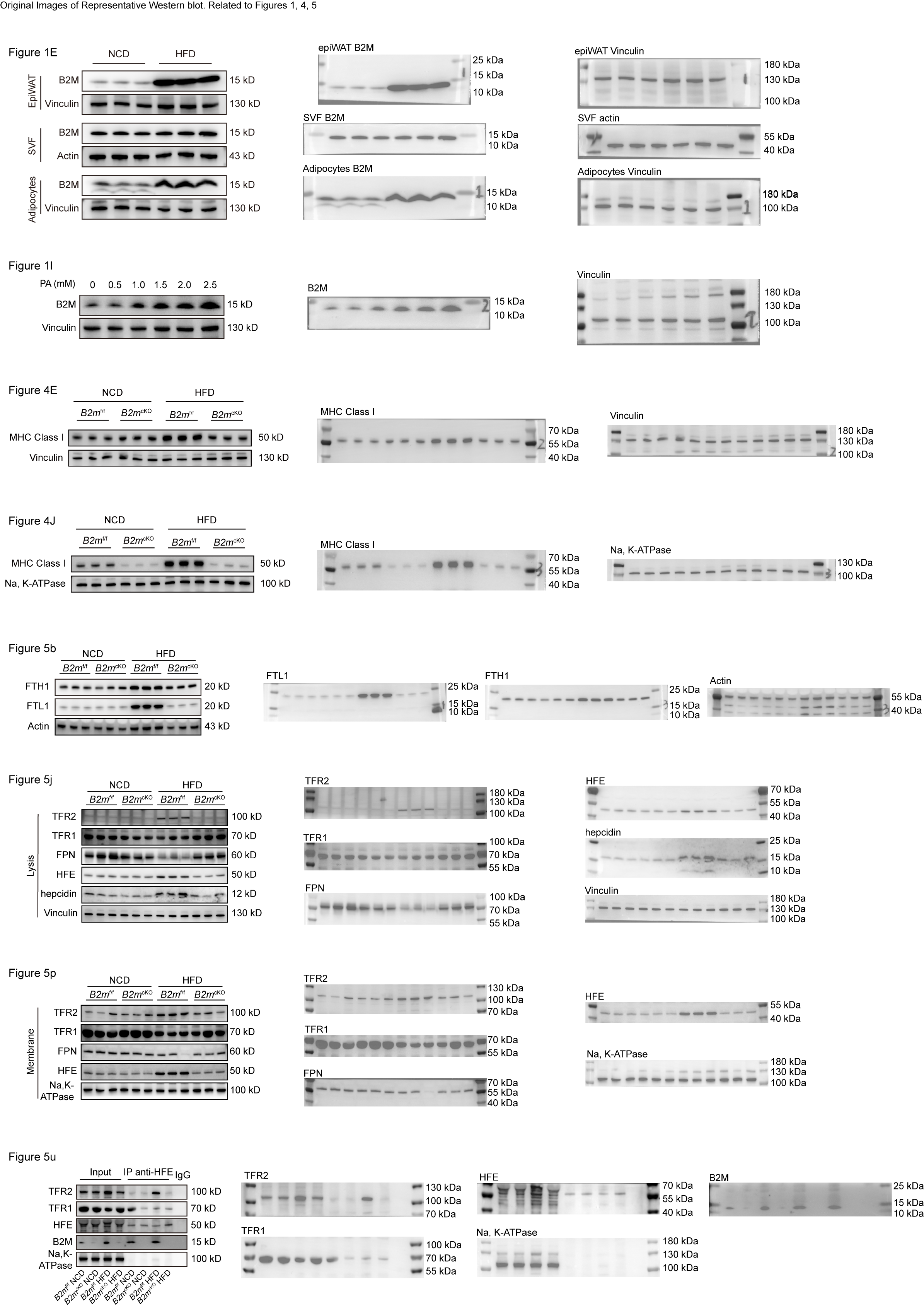

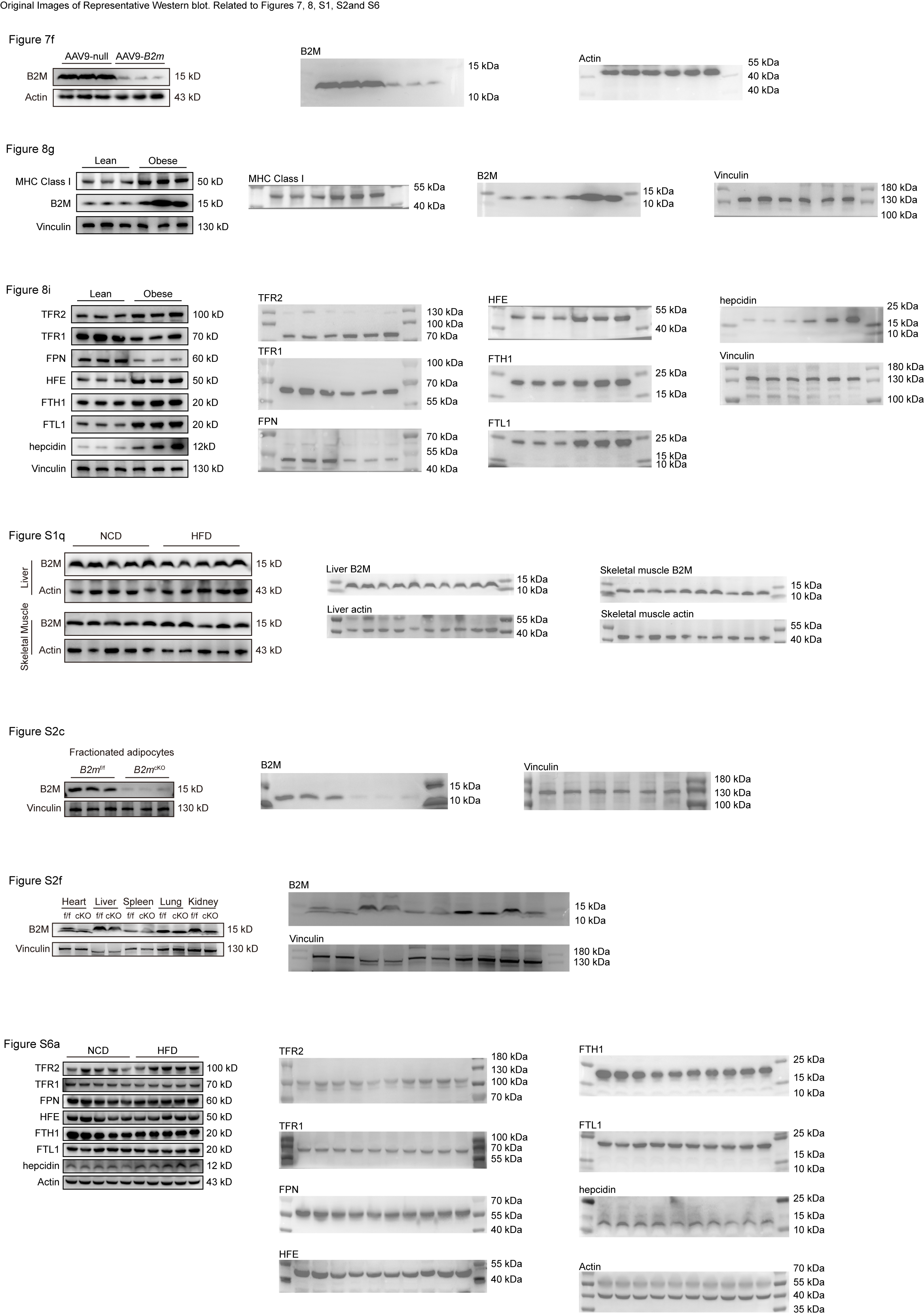

Supplement: Supplementary file 2 — Original Images of Representative Western Blot [file 41392_2025_2486_MOESM2_ESM.docx]
